# A sampling technique for worldwide comparisons of language contact scenarios

**DOI:** 10.1515/lingty-2022-0005

**Published:** 2023-02-10

**Authors:** Francesca Di Garbo, Ricardo Napoleão de Souza

**Affiliations:** University of Aix-Marseille, CNRS Laboratory Parole et Langage, Marseille, France; University of Helsinki, Helsinki, Finland; Linguistics and English Language, University of Edinburgh, Edinburgh, UK

**Keywords:** areal linguistics, areal typology, contact, diffusion, inheritance, sampling

## Abstract

Existing sampling methods in language typology strive to control for areal biases in typological datasets as a means to avoid contact effects in the distribution of linguistic structure. However, none of these methods provide ways to directly compare contact scenarios from a typological perspective. This paper addresses this gap by introducing a sampling procedure for worldwide comparisons of language contact scenarios. The sampling unit consists of sets of three languages. The Focus Language is the language whose structures we examine in search for contact effects; the Neighbor Language is genealogically unrelated to the Focus Language, and counts as the potential source of contact influence on the Focus Language; the Benchmark Language is a relative of the Focus Language neither in contact with the Focus nor with the Neighbor language, and is used for disentangling contact effects from genealogical inheritance in the Focus Language. Through this design, we compiled a sample of 49 three-language sets (147 languages in total), which we present here. By switching the focus of typological sampling from individual languages to contact relations between languages, our method has the potential of uncovering patterns in the diffusion of language structures, and how they vary and change.

## 1 Introduction

Contact between populations speaking different languages has existed throughout human history. These encounters often create scenarios of bi-/multilingualism in which linguistic features may easily diffuse (Evans 2018; Weinreich 1953). Given the crucial role that language contact plays in the history of languages (e.g., Nichols 1992), typologizing contact scenarios is a crucial step towards understanding the dynamics of language change, as well as the impact of language contact on the distribution of language structures. Yet, none of the sampling methodologies that have been used in typology are specifically tailored for comparing language contact scenarios with one another (for similar considerations see Guzmán Naranjo and Becker 2022). Although the need for studies comparing situations of language contact is often hinted at in the contact linguistics literature, where case studies abound, a comparative approach is still missing. As a result, testing generalizations about “what is common and what is not” in contact-induced change constitutes a challenging task (Backus 2014: 104). In light of this gap, the present article introduces a sampling method specifically tailored for comparisons of language contact scenarios from a typological perspective.

The proposed sampling technique departs from existing sampling methods in language typology in two ways. First, the sampling unit consists of sets of three languages rather than individual language units. Each language in the set is associated with a specific role in the current technique. The **Focus Language** is the language whose structures we examine in search for evidence of contact effects. The **Neighbor Language**, which is genealogically unrelated to the Focus Language, is the potential source of contact influence on the Focus Language. Finally, the **Benchmark Language** is a close relative of the Focus Language, which serves as a control for disentangling contact influence from genealogical inheritance in the Focus Language. Importantly, the Benchmark Language is not in contact with either the Neighbor or with the Focus Language itself. In this technique, we use evidence of contact between language communities from the literature on areal and contact linguistics to select Focus and Neighbor Languages from different regions of the world. As such, the current sample is a phenomenon-based one, with language contact being the phenomenon at stake.

The sampling method introduced here combines established practices in typological sampling with insights from both areal typology and language contact. Instead of using the geographical locations of languages as a proxy for the likelihood of contact (Dryer 1989, 1992; Guzmán Naranjo and Becker 2022; Jaeger et al. 2011; Miestamo et al. 2016; Nichols et al. 2013), the entire language selection process revolves around documented contact relations between languages. Through this design, we compiled an areally and genealogically stratified sample of 147 languages from all over the world.

As we discuss below, the procedure is easily applicable, hence offering a way of testing how linguistic structure diffuses across different contact scenarios. We argue that the use of multi-language sampling units, and the explicit inclusion of contact in the sampling technique provides a promising way of doing typological research on diverse contact settings across the world.

The paper is structured as follows. In Section 1.1, we discuss general matters related to sampling in language typology, also encompassed by our sampling technique. Section 1.2 provides an overview of approaches to language contact scenarios in areal typology, and in the language contact literature more generally. We present the details of the sampling method in Section 2, while the data set resulting from the implementation of the procedure is presented in Section 3. A discussion of contributions and limitations of the proposed technique follows in Section 4, where we also address its current and possible future applications. Section 5 concludes the paper.

### 1.1 Biases and controls in typological sampling

Put simply, sampling allows typologists to delimit the population of languages between which linguistic comparisons are drawn. This section presents a brief overview of a few fundamental principles for sampling in language typology, and how they relate to the overall goal of researching linguistic structure. For more detailed discussions of the vast literature on sampling methods, we recommend Bakker (2011), Miestamo et al. (2016), as well as Guzmán Naranjo and Becker (2022).

In principle, generalizations about the global distribution of language structures should rely on a carefully designed sampling procedure. In this respect, two of the most important desiderata in any sampling procedure are the criteria used to achieve **representativeness** and **independence**. Representativeness criteria address how a language sample reflects linguistic diversity. The criteria for independence tackle the sources of similarities between languages, be it inheritance or diffusion.

Representativeness and independence criteria may interfere with one another in various ways. For instance, representativeness may increase with the size of the sample, since a larger sample is more likely to represent the distribution of cross-linguistic patterns in the total population of human languages. At the same time, increasing sample size may compromise independence since it can inflate the likelihood of genealogical and/or areal relations between the sampled languages. One way in which researchers can address issues of representativeness and independence is to consider biases in language selection.

Widely addressed biases in typological sampling are genealogical and areal biases (for an overview of bias types in typology, see Bakker 2011).

Linguistic features such as gender systems may be particularly frequent in certain language families or areas of the world. A typological dataset that contains an overrepresentation of languages from families where given structures are common will then show a bias towards the occurrence of those very structures. For instance, sex-based gender systems are common among Indo-European as well as Semitic languages. A typological sample for a study of gender systems that is heavily skewed towards these families would inflate the frequency of sex-based systems at the expense of non-sex-based systems, which are common among Nakh-Daghestanian or Atlantic-Congo languages, for instance. Similarly, a study that investigates the distribution of gender systems, but whose sample draws heavily from Africa, would potentially over-represent the non-sex-based gender systems commonly found on that continent. In order to address these biases, typological samples tend to use stratification techniques with both genealogical and areal controls.

Stratification is a control procedure for sample overrepresentation based on genealogical and areal information. Language samples can be stratified through established genealogical groupings, and/or areal classifications. Classifications chosen for genealogical and areal controls may differ across typological samples, and may also reflect individual researchers’ goals. Given that genealogical and areal classifications function as external measures of control in typological sampling, once classifications are chosen, it is important that those criteria inform the selection of the entire dataset for a given study.

The sampling technique presented here is designed for systematic comparisons of contact scenarios on a global scale. Given that contact may be more likely to occur between geographically adjacent language communities (but see Campbell 2006: 1 for a discussion), areal stratification is a crucial step in our sampling procedure, detailed in Section 1.2.1. In order to help disentangle contact effects from genealogical inheritance, our technique focuses on sampling contact scenarios between maximally genealogically unrelated languages, that is, between languages belonging to distinct language families. Through this approach, similarities uncovered between languages in contact gain weight, given that the genealogical distance between these languages decreases the likelihood that they would show the same structures due to inheritance from a common ancestor. It follows from this consideration that genealogical stratification is another key aspect of our technique (see Section 1.2.2).

#### 1.1.1 Areality control in typological sampling

Areality is defined here as the effect of geographic proximity on the distribution of linguistic features. Areal biases affect the independence and representativeness of language samples because unrelated languages in contact may come to share a great deal of structural similarities as a result of diffusion. For instance, contact with Indo-European languages (different Germanic languages and Russian, rus) has led to changes in the syllable structure of Estonian (ekk, Uralic) (Napoleāo de Souza and Sinnemäki accepted 2022). Lack of control for areality may thus be particularly detrimental to probability samples, which aim to uncover statistical tendencies in the distribution of linguistic features. At the same time, as Bakker (2011: 95) also underscores, factoring in information about areal diffusion into typological investigations can be helpful when assessing the stability of linguistic features and their likelihood to be diffused through contact, which is one of the goals of current research agendas in distributional typology (Bickel 2010). In this section, we describe the most widely used methods to control for areality in present-day typology.

The commonest approach to areal control in typological sampling is to use continent-wide classifications as a basis for language selection. For instance, Dryer (1989, 1992 divides the world into six macro-areas,1Dryer (1989) included five macro-areas: Africa, Eurasia, Australia-New Guinea, North America and South America. In the 1989 classification, Southeast Asia was part of Eurasia and all Austronesian languages – including those spoken in New Guinea – were counted as part of Southeast Asia. In the revised version proposed in Dryer (1992), Southeast Asia and Oceania constitute an independent areal grouping, and Australia and New Guinea include all languages spoken in the area with the exception of the Austronesian languages spoken in New Guinea. roughly corresponding to major continental zones: Africa, Eurasia, Southeast Asia and Oceania, Australia and New Guinea, North America, and South America. These six macro-areas are assumed to be maximally independent from each other both in terms of language history and of contact dynamics, while also reflecting comparable levels of genealogical and typological diversity. A similar approach is taken by Hammarström and Donohue (2014), whose six-way areal classification is nonetheless based exclusively on the distribution of landmasses versus water bodies: Eurasia, Africa, Australia, Multinesia/Papunesia, North America, and South America. Hammarström and Donohue’s classification is the one currently used by Glottolog (Hammarström et al. 2020) and the latest online versions of the WALS database (Dryer and Haspelmath 2013).

**Figure 1:**
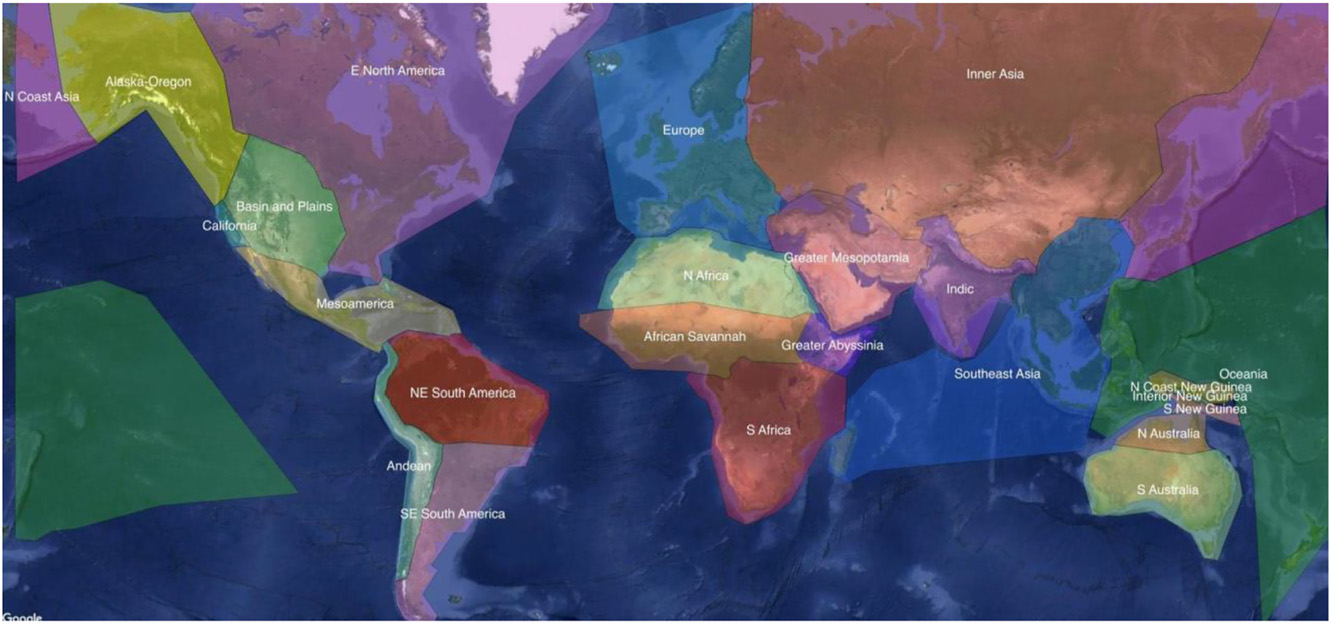
The 24 Autotyp Areas (Bickel et al. 2017, used under CC-BY 4.0 license).

A third areal classification in typology, which is also based on continent-wide areas, is the one adopted for the purposes of the Autotyp database (Bickel et al. 2022). The Autotyp areal classification consists of two levels. The first level identifies 10 wide continental areas that are roughly comparable to Dryer’s (1989, 1992 and Hammarström and Donohue’s (2014) macro-areas, but are established based on “assumptions about contact events in history, informed by current knowledge of the historical, genetic, anthropological, and archeological record” (Bickel et al. 2022). These continent-wide zones are: Africa, Western and Southern Eurasia, Northern and Central Asia, South and Southeast Asia, Australia, New Guinea and Oceania, Western North America, Eastern North America, Central America, and South America. The second level focuses on areal divisions on a smaller scale, identifying 24 different linguistic areas. These are: Alaska-Oregon, Basin and Plains, California, East North America, Mesoamerica, Andean, Northeast South America, Southeast South America, Europe, North Africa, African Savannah, Greater Abyssinia, South Africa, Greater Mesopotamia, Indic, Inner Asia, Southeast Asia, North Coast Asia, Oceania, North Coast New Guinea, New Guinea Highlands, South Coast New Guinea, North Australia, and South Australia. The 24 Autotyp Areas are shown in the Map in Figure 1. The boundaries between these smaller scale areas were established based on non-linguistic factors, such as (bio-)geography and socio-economic history (Johanna Nichols, pers. comm.). The Autotyp Areas are thus an attempt at tackling the representativeness and independence of typological samples through a procedure of areal stratification that is based on natural and cultural-economic regions of the world. The 24 Autotyp Areas have featured in typological studies investigating how the interaction of historical contingencies and cognitive abilities affect the distribution of language structures (aka ‘distributional typology’, e.g., Sinnemäki and Di Garbo 2018; Widmer et al. 2021).

Our method uses the 24 Autotyp Areas as a grid for identifying candidate contact scenarios within diverse geographical areas of the world while also providing us with a tool for areal control at a smaller scale than the continent-wide level.2Using the 24 Autotyp Areas is one way of sampling languages from within macro-areas. An alternative approach would be for instance randomly selecting languages from each continent. This procedure is discussed in detail in Section 2.

In addition to the use of pre-established geographical classifications such as those surveyed above, spatial statistics has become increasingly common in quantitative typology as a way to assess the degree of contact between speech communities. For instance, Murawaki and Yamauchi (2018) use autoregressive models to investigate the relationship between inheritance and diffusion in a selection of typological features. In a similar vein, Jaeger et al. (2011) and Guzmán Naranjo and Becker (2022) address contact by using both macro-areas and distances between languages.3However, see Gardani et al. (2021) for a discussion of the pitfalls of using geographical proximity as a proxy for language contact. While these approaches can be useful for automatic control, and to estimate the degree of contact between languages, the current technique builds samples directly based on established contact relations between language communities. We argue that this type of phenomenon-based sample is especially useful in order to test hypotheses about contact-induced change and the socio-historical correlates of typological distributions.

#### 1.1.2 Genealogical control in typological sampling

Since structural similarities between languages often result from inheritance, all language sampling techniques rely on some principled strategy for genealogical control. Indeed, as Guzmán Naranjo and Becker (2022: 628) highlight, genealogical control may even be prioritized at the expense of areal control. Genealogical stratification increases the independence of data points and thus the representativeness of typological data sets with respect to patterns of linguistic diversity. This kind of stratification is achieved by choosing a genealogical classification as a reference point, and further specifying the level of classification to select languages from (e.g., the family or subfamily level), as well as the number of languages sampled per genealogical level (see Bakker 2011, and Miestamo et al. 2016 for reviews). In order to control for genealogical biases, some studies sample genera4A genus is a level of classification with a time-depth spanning between 3,500 and 4,000 years. The choice of 3,500–4,000 years as a cut-off point is primarily based on the proposed time-depth of the different sub-branches of the Indo-European family such as Celtic, Germanic, Indo-Aryan, Italic, and so on. The approach rests on the observation that, within such groupings, languages tend to be typologically quite similar to each other. Moreover, controversies in language classification most often lie at deeper time depths. For instance, comparative reconstructions stretching beyond 3,000–4,000 years are less readily verifiable through the comparative method (Dryer 1989: 286). instead of languages (Bickel 2008; Dryer 1989, 1992). Other studies include genus or family in a statistical model, either as random effects (Jaeger et al. 2011) or fixed effects (Bickel 2008). Yet another approach is to use phylogenetic regression to account for genealogical dependencies between sampled languages (Guzmán Naranjo and Becker 2022; Verkerk 2013).

Alongside areal stratification, genealogical control features prominently in the sampling technique presented in this article, given that we only study contact relations between genealogically unrelated languages. In order to identify pairs of unrelated languages in contact, we use the genealogical classifications in Glottolog 4.2.1 (Hammarström et al. 2020) as a starting point. We remain neutral to the analyses underlying those classifications, and acknowledge that these analyses may be subject to change based on the progress of historical-comparative reconstruction of given language groupings. The criteria that we use in order to operationalize Glottolog’s families into the proposed sampling technique are described in detail in Section 2.

### 1.2 Establishing and comparing contact scenarios: insights from areal typology, areal linguistics, and contact linguistics

Areal typology and areal linguistics investigate the processes through which languages in contact may become similar because of interactions between neighboring communities. However, what characterizes studies in areal typology vis-à-vis areal linguistics is the focus on areal effects from a cross-linguistic perspective (cf. Dahl 2001: 1456). Areal typology has gained ground over the past 20 years due to a growing interest in disentangling functional-communicative pressures from socio-historical dynamics in typological distributions (Bickel 2010; Koptjevskaja-Tamm 2011).

Areal studies typically conceive of contact influence as resulting from **bundles of languages in interaction**. Linguistic outcomes of contact within these bundles are generally distributed along multiple trajectories, which can be depicted in the form of isoglosses. Yet another approach characterizes contact studies, which typically investigate language contact from the perspective of individual languages, and are mostly based on case studies. In this context, contact influence is described as resulting from interactions between **pairs of languages in contact**.

Our procedure attempts to reconcile the research traditions of areal and contact studies. It relies on geographical classifications that take in to account linguistic geography and areality. At the same time, it constructs sampling units in a pairwise fashion, based on a common practice in language contact research. As outlined in Section 2 and subsequently demonstrated in Section 3, this approach results in a sampling tool that is easily applicable to a variety of contact scenarios beyond those that we have sampled for the purpose of this demonstration.

## 2 Sampling procedure

We based the selection of the three-language sets on four criteria: (1) geographical area; (2) independently reported contact scenarios; (3) genealogical distance between languages in contact; and (4) availability of reference materials for each of the languages in the set. These four criteria enable us to build a typological dataset that is geographically and genealogically stratified, in addition to being independent of our own assessment of a given contact situation.

As mentioned in Section 1, the sampling procedure presented here uses three-language sets rather than individual languages as the sampling unit. Each set contains a Focus Language, a Neighbor language, and a Benchmark Language. The role of Focus or Neighbor Language is assigned based on resources describing individual contact scenarios,5Of course, a language can be in contact with multiple others at any given time, as is known for many linguistic areas of the world (see Muysken 2007, as well as Pakendorf et al. 2021 for scenarios of small-scale multilingualism). Still, as our procedure shows, one can study contact in pairs of ‘Focus’ and ‘Neighbor’ languages even in highly multilingual contact scenarios. This approach has the advantage of being applicable both to pairwise (e.g., English [eng] and Welsh [cym] in Wales, UK) and multiple language interactions (e.g., Meyah [mej], Moi [mxn], Hatam [had], Mpur [akc], etc. in West Papua, Indonesia). and on the existence of related languages for the Focus (see below). Importantly, the current technique is meant for studies that evaluate the extent to which contact influences the structures of Focus Languages, rather than serving as a starting point for determining whether or not contact has indeed existed.6The use of the terms ‘Focus’ and ‘Neighbor’ here also highlights the fact that Focus Languages may in turn influence structures of Neighbor Languages, given that linguistic structure may diffuse both ways. Alternative labels such ‘donor’ versus ‘replica/recipient language’, or ‘superstrate’ versus ‘substrate language’ (Winford 2010: 171), among others, may imply a unidirectional approach.

The third language of our sampling unit is the Benchmark Language, and serves as a parameter against which to test the impact of contact on the Focus language. The Benchmark Language is as closely related to the Focus Language as possible, while at the same time not being in contact with either the Focus nor the Neighbor Language (see Section 2.3). In our technique, Benchmark Languages provide a means of assessing what structural features of the Focus Language may be due to diffusion rather than inheritance. That is, we can interpret structures of the Focus Language that systematically differ from those present in the Benchmark, while mirroring properties of the Neighbor Language, as evidence for contact (see Section 5 for examples). For a similar approach in sociolinguistics, see Torres Cacoullos and Travis (2018).

In order to fulfill the specific goals of our own ongoing project, we used a final, project-specific criterion. Since part of this research aims to produce sociolinguistic descriptions of the contact dynamics between Focus and Neighbor languages, we established that there should also be living experts on the Focus Language with whom we could collaborate. This additional criterion will be briefly discussed in Section 5 since it has no bearing on the sampling procedure. The remainder of this Section deals with the four other criteria cited above.

### 2.1 Geographical area

This first criterion allows us to draw a sample that represents contact scenarios from all parts of the world. It thus complies with general representativeness desiderata in typological sampling (Section 1.2). Given that one of the goals of our methodology is to generate a sample for testing contact effects on linguistic variables, we opted for using a geographical classification that is at least in part delimited on criteria other than linguistic ones (Section 1.2.1). Thus, the 24 areas established for the Autotyp database (Bickel et al. 2022) form the basis for areal stratification.

The Autotyp Areas differ from other geographical classifications in that they derive from a combination of archeological, anthropological, historical, and genetic data rather than from linguistic features alone (Nichols et al. 2013: 6). The 24 Areas in the Autotyp classification (Figure 1) also present the advantage of zooming in onto continent-wide areas, which is not the case in any of the other widely used classifications in typology, such as the one adopted by Glottolog. This classification thus provides us with a grid to pursue representativeness within continent-wide areas. Table 1 lists the areas we used in the current technique in comparison to (a) continental landmasses, and (b) the Glottolog areas.

**Table 1:** The Autotyp Areas by continental landmass, and as compared to Glottolog areas.

Continental landmass	(N)	Autotyp Areas	Glottolog areas
Africa	(4)	African Savannah Greater Abyssinia North Africa Southern Africa	Africa
Eurasia	(6)	Europe Greater Mesopotamia Indic Inner Asia North Coast Asia Southeast Asia	Eurasia
North America	(5)	Alaska-Oregon Basin & Plains California Eastern North America Mesoamerica	North America
Oceania	(6)	North Australia South Australia Interior New Guinea North Coast New Guinea Southern New Guinea Oceania	Australia Papunesia
South America	(3)	Andean Northeast South America Southeast South America	South America

Initially, we aimed at selecting two sets from each of the 24 Autotyp Areas (i.e., 2 sets × 3 languages × 24 areas = 144 languages). In order to arrive at an even 150 languages,7Setting the sample size to 50 sets, or 150 languages, partly derives from methodological necessities related to our project-specific goals. It constitutes a compromise between having enough data to investigate the typological distribution of various language structures in the languages of the sample, and maintaining a manageable workflow within the timespan of the project at large. For an illustration of the breadth and depth of the ongoing linguistic data collection in the context of the larger project, see Section 4. we decided to include two extra sets, one from Northeast South America, and the other from the North Coast of New Guinea. We selected South America and New Guinea as the sources for the two additional sets because of the high degree of linguistic diversity found in those areas as a whole (e.g., Dahl 2008; Hammarström 2016). For reasons explained in Section 2.2, only one set could be sampled for Southern Australia, which yielded a final sample of 49 sets and 147 languages in total.

### 2.2 Independently reported contact scenarios

This second parameter guides the choice of Focus and Neighbor Languages to analyze within each Autotyp Area. To fulfill this criterion, we relied on descriptions of contact scenarios in the language contact and areal linguistics literature, primarily through macro-area surveys of contact situations. For instance, we drew our Northeast South America sets based on the surveys of contact in the Amazon Region by Aikhenvald (2002, 2012. From those surveys, we then selected the ‘Vaupés Area’ as a starting point from which to draw specific contact scenarios and individual languages.

Descriptions of individual cases of contact varied in detail across the different areas. For instance, some language contact situations have a longer history of study, such as the Pacific Northwest in North America (Thompson 1979), whereas others stem from more recent proposals, for instance those involved in the Mamoré-Guaporé Area in South America (Crevels and van der Voort 2008). Whenever possible, we opted for contact scenarios with multiple instances of documentation in the literature. Table 2 illustrates some of the materials we consulted to arrive at our individual sets.

**Table 2:** Examples of areal and contact linguistics surveys used as primary sources to draw contact scenarios for the sample, shown in alphabetical order by editor’s last name.

Reference material	Author(s)	Used for the Autotyp Area(s)
*Language contact in Amazonia*	Aikhenvald (2002, 2012	Northeast South America
*The Oxford handbook of language contact*	Grant (2020)	Alaska-Oregon, Southeast Asia, Greater Mesopotamia, Indic
*The languages and linguistics of Africa*	Güldemann (2018)	North Africa, Greater Abyssinia, Southern Africa
*The handbook of language contact*	Hickey (2010)	Inner Asia, North Coast Asia, California
*The Cambridge handbook of areal linguistics*	Hickey (2017)	Greater Mesopotamia, Southeast Asia, Southeast South America
*The languages and linguistics of New Guinea*	Palmer (2018)	Southern New Guinea, North Coast New Guinea, Interior New Guinea, Oceania

The task of matching contact scenarios with the Autotyp Areas was straightforward in most cases. Whenever we were uncertain where to place a case within an Autotyp Area, we relied on the geographical location of the Focus and Neighbor languages as stated in Glottolog. In a few cases, we also consulted specialists in the specific contact situations. A few Autotyp Areas presented challenges of their own. These are the three Autotyp Areas in the island of New Guinea, the North Coast Asia Area, Eastern North America, and Southern Australia.

The New Guinea Areas, and North Coast Asia are all relatively small, so that finding a Benchmark proved especially challenging in some cases. Additionally, New Guinea shows less in-depth documentation given the linguistic diversity in the region compared to other parts of the world. Eastern North America showed high degrees of language obsolescence, which had some impact on sampling composition (see Section 5). Finally, the spread of the Pama-Nyungan family in Southern Australia posed an insurmountable problem to the application of the selection criteria. The ubiquity of Pama-Nyungan led to a very reduced choice of contact scenarios between genealogically unrelated languages in that part of the world. As a result, only one set was sampled for the Southern Australia Autotyp Area.

We wish to underscore that aside from selecting sources that provided a detailed description of the contact scenario from a linguistic standpoint, we made no further evaluations as to the nature of the contact relations described in the literature. As such, we remain neutral to any disagreements or disputes over the plausibility of individual proposals, especially regarding larger linguistic areas. Finally, neither the type of contact profile instantiated by the candidate contact scenarios (e.g., regarding ‘intensity’ or ‘symmetry’), nor the linguistic outcomes of contact reported in the literature played any role in the selection procedure.

### 2.3 Genealogical distance

In order to minimize possible confounds due to common ancestry, we established that each set in our sample would include only Focus and Neighbor Languages that belonged to distinct language families. This third criterion helps us ensure that linguistic effects on Focus Languages stem from the contact situation rather than from shared inheritance (but see how the current technique would fare in studies of more closely related languages in Section 4). Genealogical distance thus represents a way to address the independence desideratum in typological sampling (Section 1.2).

As previously mentioned, we used the classifications in Glottolog as a parameter for genealogical distance between Focus and Neighbor Language within a given set. For the purposes of this paper, a language family corresponds to the highest level of genealogical classification in Glottolog (i.e., “top-level family”), meaning that a grouping such as ‘Finnic’ constitutes a subfamily in our classification, rather than a family (in this case, Uralic). All Glottolog classifications used in this paper refer to the database as of April 2020 (i.e., version 4.2.1; Hammarström et al. 2020).

For instance, applying this criterion to the contact scenarios in the Vaupés Area gave us the option of selecting the Naduhup language Yuhup (yab) as the Focus language, and a Tucanoan language such as Macuna (myy) as the Neighbor Language (Aikhenvald 2002). Determining which language should be the Focus, and which should be the Neighbor Language often hinged on the level of detail in the description of contact, and on the availability of reference materials (see Section 2.4). Additionally, in a few cases discussed below, the internal structuring of a given language family influenced sample composition, especially regarding the choice of the Benchmark Language. For each Focus Language, a Benchmark Language was chosen from among the Focus Language’s closest relatives8This criterion naturally excludes language isolates from our choice of Focus Languages, since isolates have no relatives. Language isolates may however figure among Neighbor Languages in our technique, as is the case in a few of our sampling sets (e.g., Basque, Karok, and Zuni in Table 3, see also Appendix A in the Supplementary Materials). as stated in Glottolog 4.2.1 (Hammarström et al. 2020).

The choice of Benchmark poses a challenge in itself, since two closely related languages may indeed be involved in the same contact scenario.9Another related challenge concerns language families that are spread over large geographical areas. In some such cases, Benchmark Languages might themselves be in contact with a relative of the Neighbor Language, even if spoken outside the Focus-Neighbor contact zone. While we are aware that this possibility might weaken the role of the Benchmark as an external source of control, it remains to be seen the extent to which such cases occur. Such a possibility led us to establish further criteria for the selection of Benchmark Languages. First, the Benchmark Language must not be part of the Focus/Neighbor contact zone. Ensuring that the Benchmark language is not subject to the same contact influences as the Focus language strengthens its status as a source of external control. When more than one candidate was available, we chose the one that was geographically closest to the Focus Language. For the current sample, we relied on the sources described in Section 2.1.2 to rule out the Benchmark Language’s participation in the contact scenarios we sampled. More specifically, we double-checked reference materials for mentions of contact between Benchmark, Focus and Neighbor Languages, and replaced any Benchmarks that participated in contact scenarios with either FL or BL.10It is evidently impossible to rule out sporadic contact that may have gone unnoticed by authors of reference materials, as well as contact in the remote past. Based on the sources, however, we must conclude that any such events might have had a negligible effect on the languages in question, if at all. Contact between the Benchmark and other languages, possible as they may be, played no role in the selection process.

**Figure 2:**
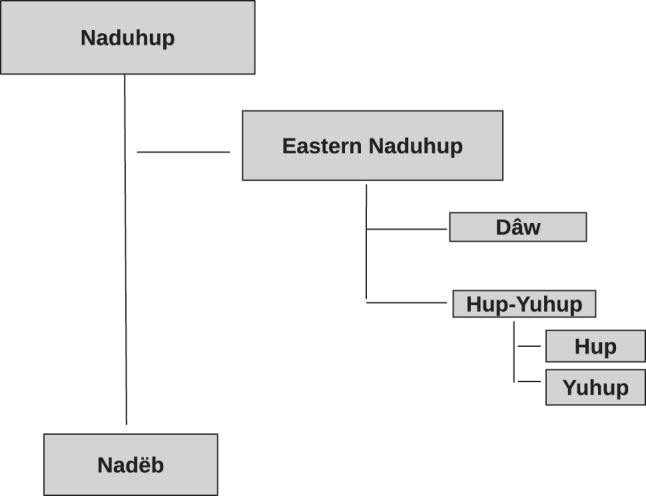
The Naduhup family tree based on Glottolog (Hammarström et al. 2020).

**Figure 3:**
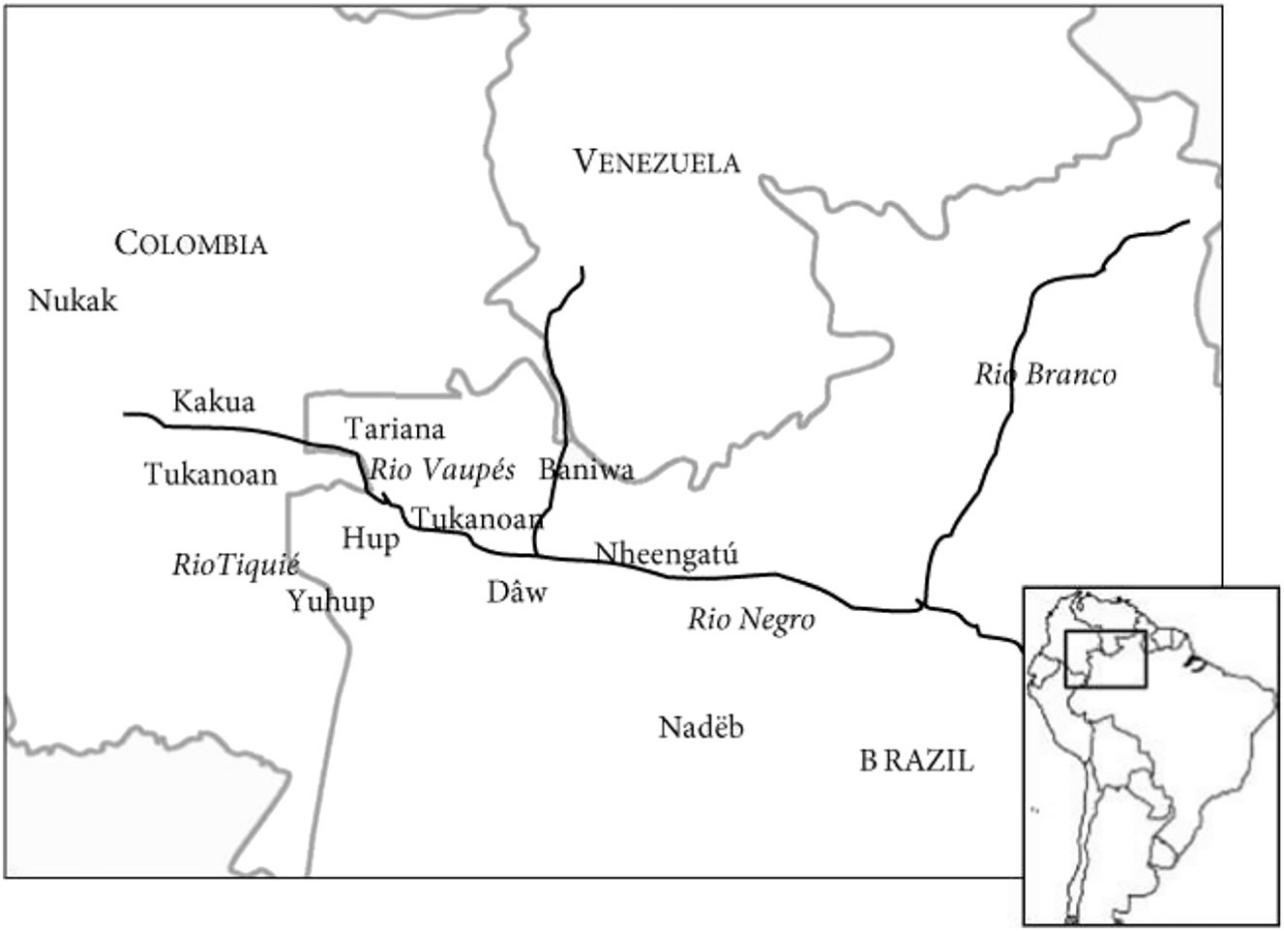
The Naduhup languages with respect to the other languages of the Vaupes region. Map taken from Epps (2013: 333), available Open Access. Scale: 1 cm–100 km (our estimate).

The Vaupés contact zone once again serves as an illustration. Following the steps outlined above, we find that Yuhup’s closest relative is Hup (jup), which also belongs to the Hup-Yuhup sub-branch of the Naduhup family in the Glottolog classification. Since Hup also participates in contact scenarios involving languages in the Tukanoan family, possibly with the Neighbor Language Macuna itself, selecting Hup violates one of the criteria for selection as Benchmark. The next closest relative to Yuhup would then be Dâw (kwa). However, upon double-checking our references, we found that Dâw too played a role in this particular contact scenario. As such, we moved on to the next relative, finally selecting Nadëb as the Benchmark Language in the Hup-Macuna set. Figure 2 illustrates the genealogical relations between the languages of the Naduhup family according to Glottolog. The location of Hup, Yuhup, and Nadëb with respect to the other language families of the Vaupés is shown in Figure 3.

### 2.4 Availability of reference materials for linguistic data collection

This criterion relates to the overall goals of our larger project, namely to test the effects of contact on a range of linguistic variables. Our research aims to compare given variables in the Focus Language to those of the Neighbor Language, using the Benchmark Language as a parameter for comparison. Thus, one relevant caveat that this method introduces is that each sampling unit requires three different descriptions of appropriate quality for the languages in each set. Reference materials such as grammars, journal articles, and dictionaries were deemed appropriate for the goals of our study. While we may use additional sources such as databases and data from conference presentations, we established that the three languages in each of the sets must have primary linguistic sources for them to be included in the sample.

Selecting reference materials for Benchmark Languages was a relatively uncomplicated task given that there were often different options for each set. On the other hand, the lack of appropriate descriptions for Neighbor Languages posed a much greater challenge. In many cases, the Focus Language was in contact with a single language belonging to a different family, so that the lack of materials for that particular Neighbor Language led to the exclusion of the entire set. In cases for which there were two or more candidate Neighbor Languages, we selected the one we deemed had the most suitable description. As discussed in Sections 4 and 5, in some cases, the choice of Neighbor Language was narrowed down through discussions with experts of the sampled contact scenarios.

## 3 Results

This section describes the sample of 147 languages obtained following the criteria explained above. In short, the central goal of our procedure is to arrive at a sample of language contact scenarios from all parts of the globe. Our sampling unit consists of three-language sets: a Focus Language, a Neighbor Language, and a Benchmark Language. The Focus and Neighbor languages are in direct contact, and they belong to distinct language families. The Benchmark Language is related to the Focus Language, but is not in contact with either the Focus or the Neighbor languages.

**Figure 4:**
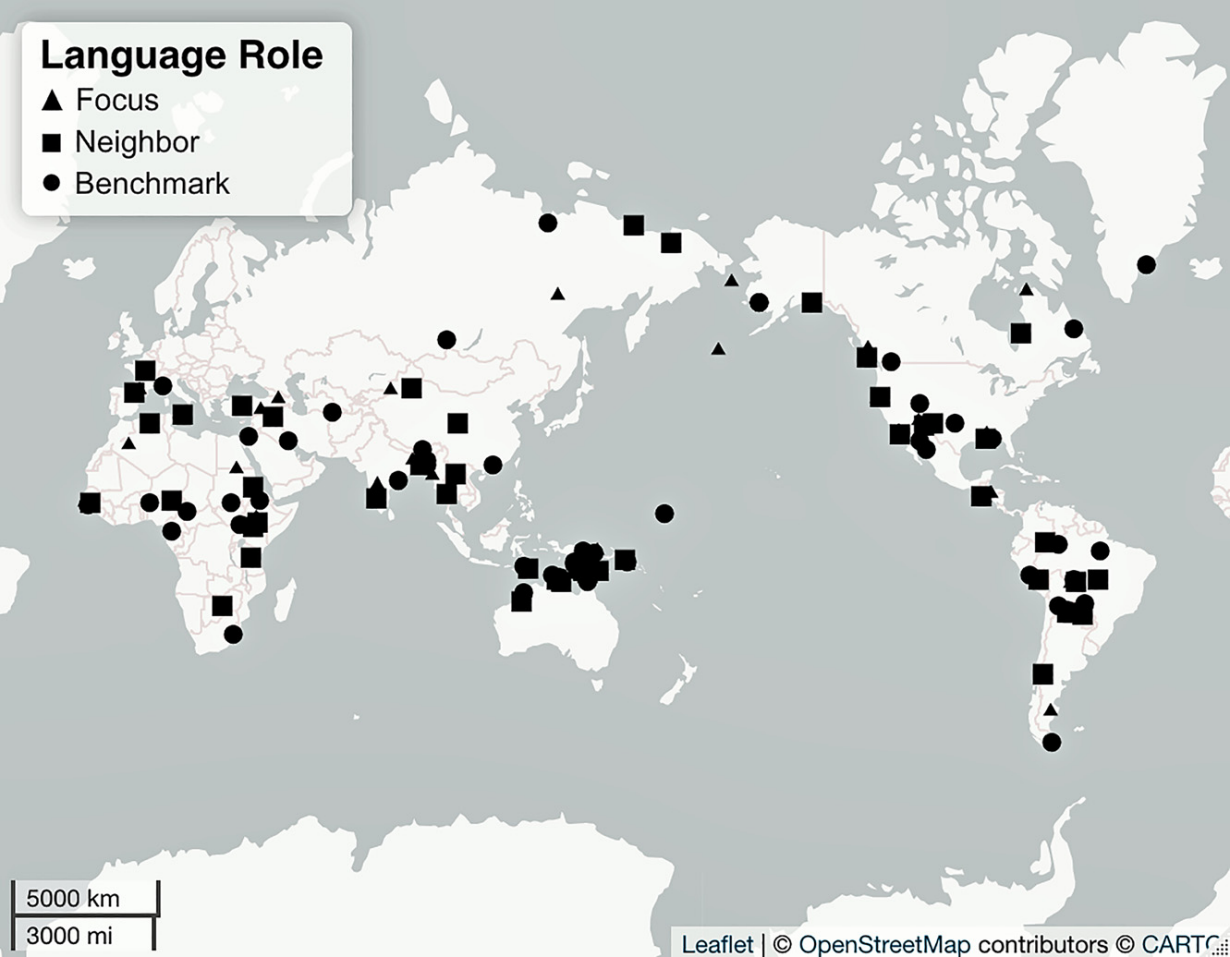
The language sample (for illustrative purposes).

**Figure 5:**
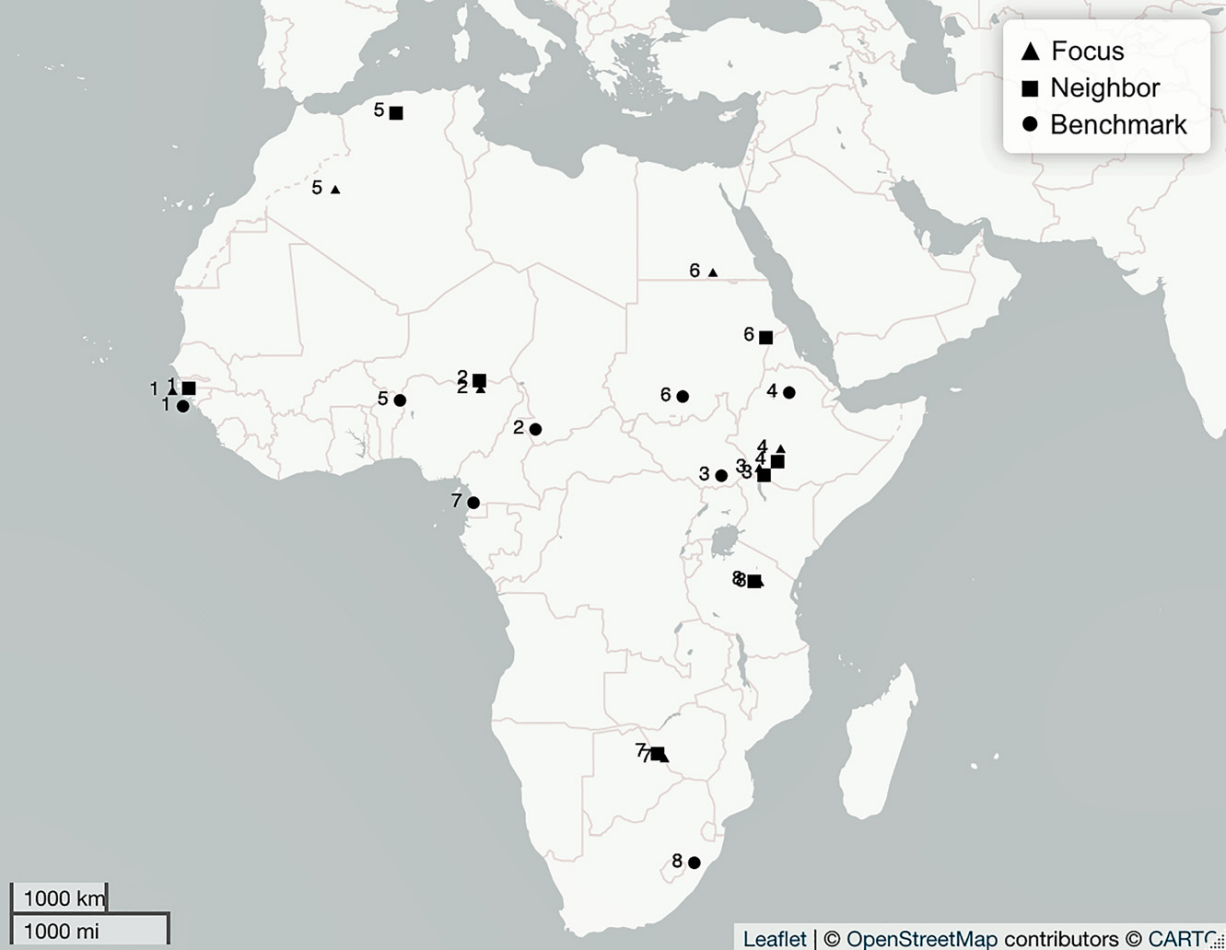
The Africa sets by Set ID and Autotyp Area (see Appendix A, Supplementary Materials).

**Figure 6:**
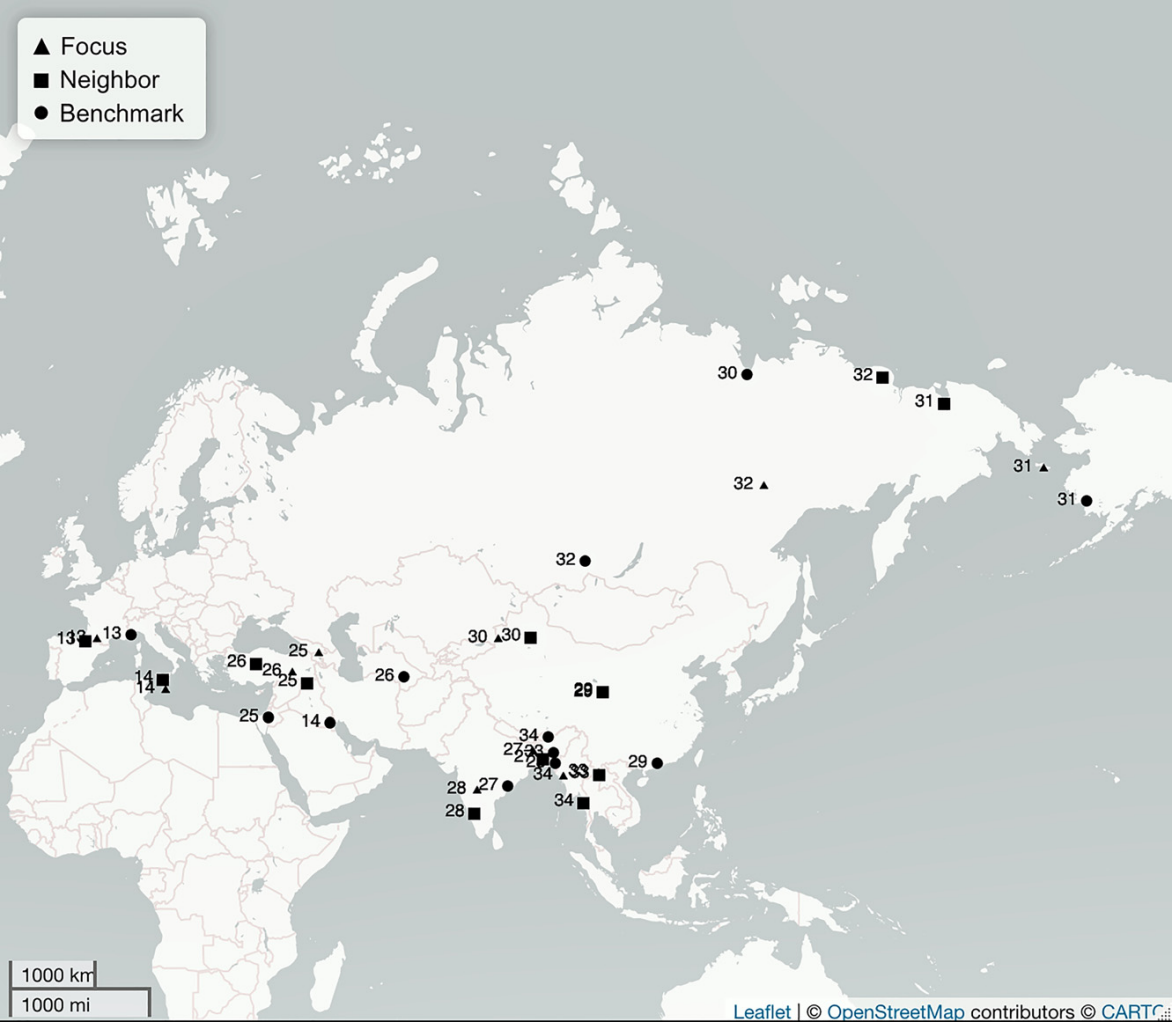
The Eurasia sets by Set ID and Autotyp Area (see Appendix A, Supplementary Materials).

**Figure 7:**
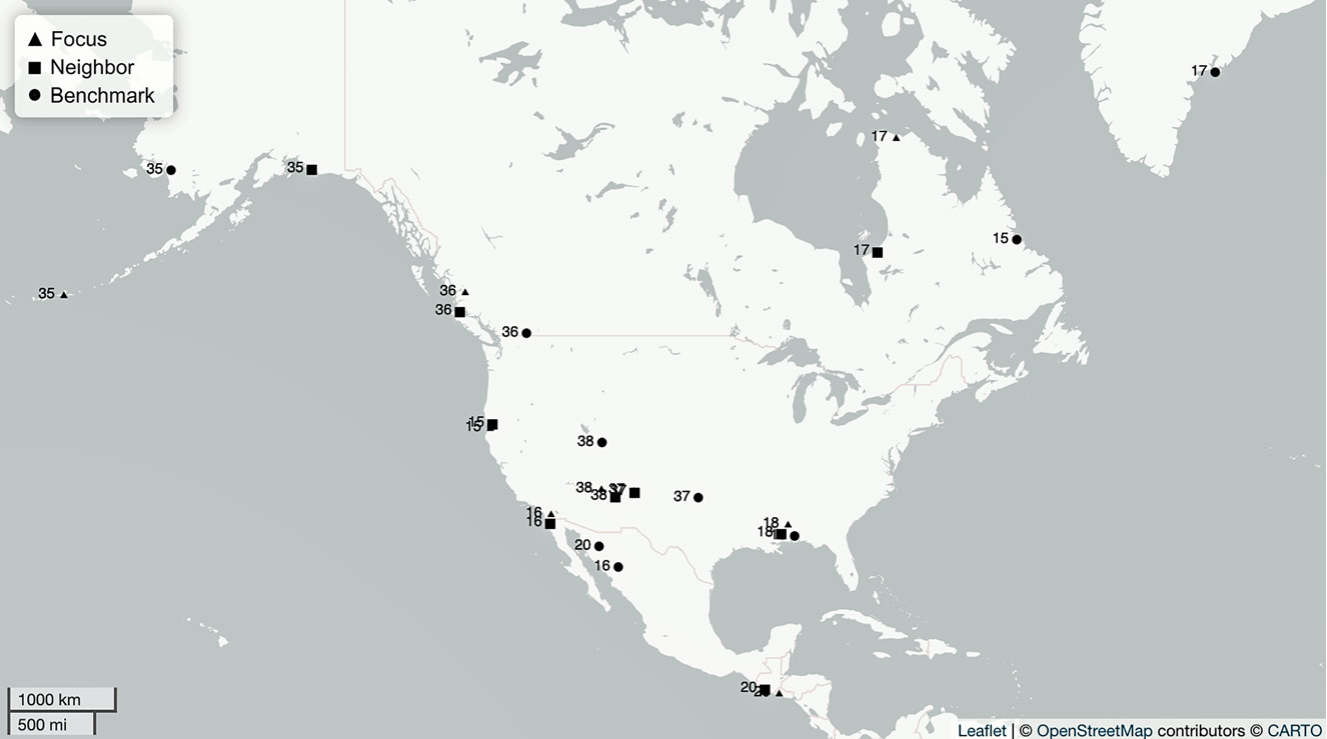
The North America sets by Set ID and Autotyp Area (see Appendix A, Supplementary Materials).

**Figure 8:**
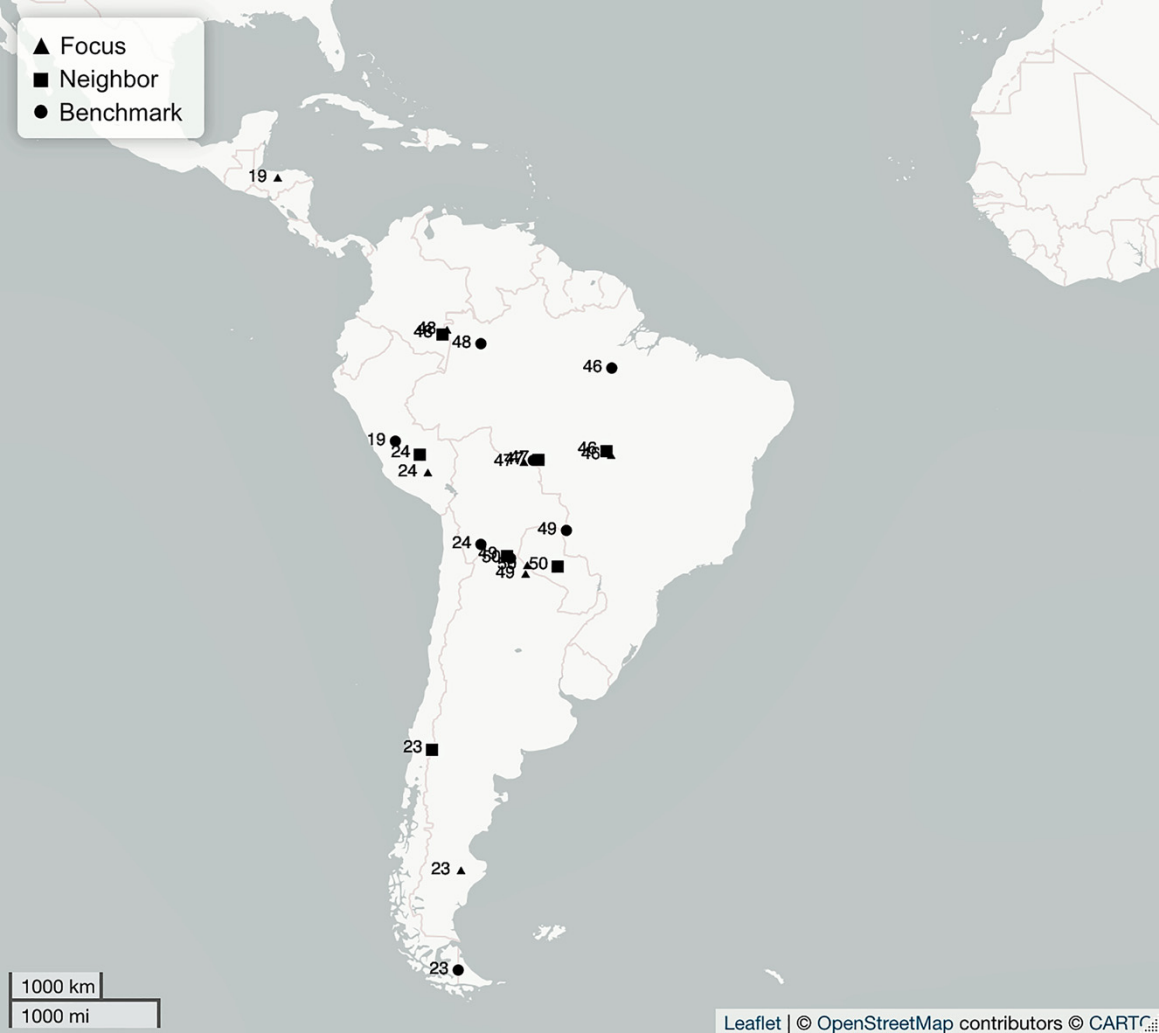
The South America sets by Set ID and Autotyp Area (for details, see Appendix A, Supplementary Materials).

**Figure 9:**
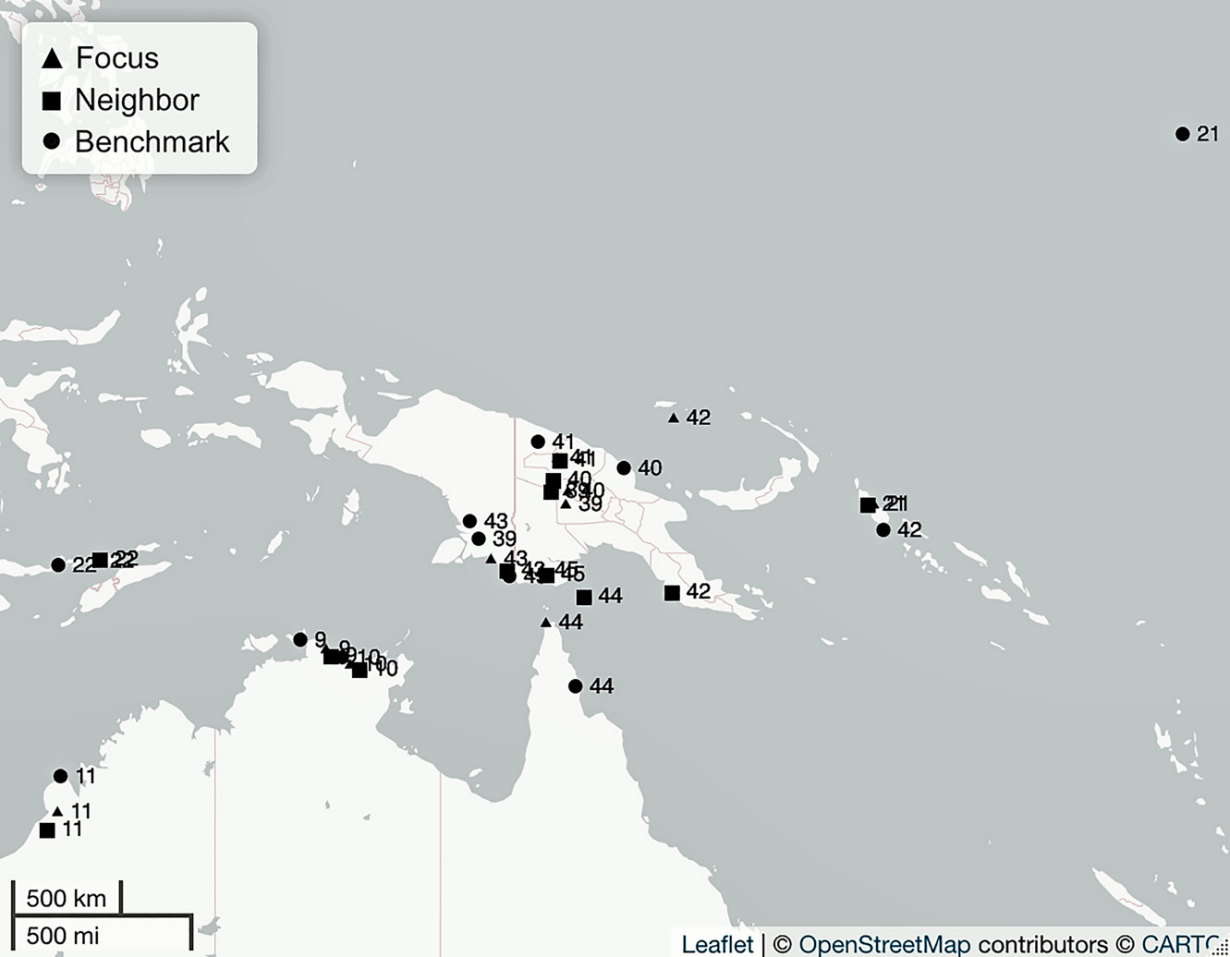
The Australian and Papunesia sets by Set ID and Autotyp Area (for details, see Appendix A, Supplementary Materials).

We successfully applied the sampling methodology described above matching language selection against the four criteria introduced in Section 2. We identified suitable contact scenarios between languages of different families from various parts of the world (criteria 1, 2, and 3), and we also found source materials for all languages of all 49 contact scenarios (criteria 4). Table 3 illustrates 10 of our sets while the full sample is presented in Appendix A in the Supplementary Materials. Figure 4 illustrates the distribution of the sampled Focus, Neighbor, and Benchmark languages across the world. Figures 5–9 zoom in on continental areas (i.e., Africa, Eurasia, North America, South America, Australia and Papunesia). Geographical locations for each of the sampled languages were extracted from Glottolog using the R package lingtypology (Moroz 2017). Glottolog’s coordinates are based on the geographical center-point of the area where the speakers of a language live nowadays. At times, these coordinates may also reflect historical locations, a demographic center-point, or other representative point for a given language community.

**Table 3:** Selected examples of language sets.

Set	Continental mass	Autotyp Area	Focus Language (ISO; Family^a^)	Neighbor Language (ISO; Family^a^)	Benchmark Language (ISO; Family^a^)
04	Africa	Greater Abyssinia	Kambaata (ktb; AFA)	Wolaytta (wal, TNO)	Xantanga (xan, AFA)
07		Southern Africa	Ndebele (nde, ACG)	Tjwao \(tjwa1234, KKW)	Gyele (gyi, ACG)
13	Eurasia	Europe	Gascon Occitan (oci; IEU)	Basque (eus; EUS)	Ligurian (lij; IEU)
27		Indic	Santali (sat; ATC)	Bengali (ben; IEU)	Gata’ (gaq; ATC)
09	Oceania	North Australia	Mawng (mph; IWA)	Kunbarlang (wlg; GGN)	Iwaidja (ibd; IWA)
21		Oceania	Papapana (ppn; AUN)	Rotokas (roo; NOL)	Marshallese (mah; AUN)
15	North America	California	Yurok (yur; ALG)	Karok (kyh; KYH)	Naskapi (nsk; ALG)
38		Basin and Plains	Hopi (hop; UAZ)	Zuni (zun; ZUN)	Ute-South Paiute (ute; UAZ)
24	South America	Andean	Cusco Quechua (quz; QUE)	Machiguenga (mcb; AWK)	South Bolivian Quechua (quh; QUE)
48		NE South America	Yuhup (yab; NHP)	Macuna (myy; AWK)	Nadëb (mbj; NHP)

^a^Language family abbreviations are listed in Appendix B, Supplementary Materials.

For all of the 49 sampled contact scenarios, the criteria proposed led to the successful identification of pairs of Focus and Neighbor languages. This was the case also in highly multilingual areas of the world, such as the US-Canada Pacific Northwest, or in the Mamoré-Guaporé area. In some cases, the criterion establishing that the Focus and Neighbor Languages must belong to separate language families allowed us to select a single pair, even in those multilingual settings. For instance, in the Pacific Northwest, we selected Salishan Nuxalk (blc) and Wakashan Kwakw’ala (kwk), and for the Mamoré-Guaporé Area, we picked Tupian Mekens (skf) in contact with the isolate Aikanã (tba). In other cases, we selected the two languages whose speakers had been in contact *the longest*, according to our sources. This was the case for the Northern Australia scenario involving Mawng (mph) as the Focus, and Kunbarlaang (wlg) as the Neighbor Language.

Lastly, even though we did not explicitly control for family representation across the sampled sets, the method yielded a diverse sample of language families, with a total of 64 families. The sampled language families, and the number of Focus and Neighbor languages sampled for each of them are given in Table 4. As the Table shows, the majority of language families are represented only once in the sample (either as Focus or as Neighbor languages, e.g., Anim or Saharan). Some families (e.g., Arawak or Algic) are represented once in the Focus and once in the Neighbor language role. Finally, language families that are spread across wide areas (e.g., Afro-Asiatic or Uto-Aztecan) are represented more than once in the sample, albeit in different roles within sets.

**Table 4:** Number of Focus (FL) and Neighbor Languages (NL) by language family. The total number of language families in the sample is 64.

Language family	FL	NL	Language family	FL	NL
Atlantic-Congo	3	–	Muskogean	1	–
Athabaskan-Eyak-Tlingit	–	1	Mayan	–	1
Afro-Asiatic	4	3	Ndu	–	1
Aikanã	–	1	Naduhup	1	–
Algic	1	1	North Bougainville	–	1
Anim	1	–	Nuclear-Trans-New-Guinea	2	–
Araucanian	–	1	Nubian	1	–
Austroasiatic	2	1	Nyulnyulan	1	–
Austronesian	3	–	Pahoturi	–	1
Arawak	1	1	Pama-Nyungan	1	2
Basque	–	1	Quechuan	1	–
Cochimi-Yuman	–	1	Saharan	–	1
Chonan	1	–	Sepik	1	1
Chukotko-Kamchatkan	–	1	Songhay	1	–
Cariban	1	–	Salishan	1	–
Dravidian	–	1	South Omotic	–	1
Duna	–	1	Sino-Tibetan	2	–
Eskimo-Aleut	3	–	Surmic	1	–
Eastern Trans-Fly	–	1	Siouan	–	1
Guaicuruan	1	–	Tai-Kadai	–	1
Gunwinyguan	–	1	Tucanoan	–	1
Indo-European	3	5	Timor-Alor-Pantar	–	1
Iwaidjan	1	–	Ta-Ne-Omotic	–	1
Keresan	–	1	Tupian	1	–
Khoe-Kwadi	–	1	Trumai	–	1
Kiowa-Tanoan	1	–	Tungusic	1	–
Karok	–	1	Turkic	1	3
Lengua-Mascoy	–	1	Uto-Aztecan	3	–
Mande	–	1	Wakashan	–	1
Mongolic-Khitan	–	1	Yam	1	–
Maningridan	1	–	Yukaghir	–	1
Marori	–	1	Zuni	–	1
Matacoan	1	1			

## 4 Discussion

In this section, we outline the contribution of our method to the study of linguistic diversity (Section 4.1) and contact effects (Section 4.2). In Section 4.3, we discuss the limitations of the method and how to address them. The current implementation of the procedure and possible further applications are discussed in Sections 4.4 and 4.5, respectively.

### 4.1 Applicability of the sampling procedure

The dataset generated through our sampling procedure fully complies with the four criteria introduced in Section 2, namely: geographical area, independently reported contact scenarios, genealogical distance, and availability of reference materials. By using the Autotyp Areas and Glottolog classification as bases for areal and genealogical control, our dataset meets key desiderata in typological sampling, in that it is both geographically and genealogically stratified. Moreover, by selecting two-three contact scenarios per Autotyp Area, the method also achieves representativeness in that it includes a diverse range of sociolinguistic contexts from various parts of the world (Section 1).

The sampling procedure utilizes insights from areal linguistics, areal typology, historical linguistics, and language contact research, being further guided by the contact literature. This method makes one important contribution to sociolinguistic typology (Croft 2021; Trudgill 2011). It is the first technique of its kind to allow for worldwide comparisons of contact scenarios, one of the key goals of the sociolinguistic typology enterprise. While there have been other attempts to study contact from a cross-linguistic perspective, and from the perspective of quantitative typology, (e.g., Chang and Michael 2014; Murawaki and Yamauchi 2018), the novelty of our approach resides in providing a sampling method that builds directly on established contact scenarios, which in turn allows for conducting large-scale comparisons of language contact phenomena.

### 4.2 The procedure regarding the study of contact effects

The sample presented here is currently being used to gather linguistic data in the context of our larger project (see Section 1). We are coding for 200 different variables in the realms of phonology, morphosyntax, and lexicon in each of the languages in the sample. More specifically, the coding encompasses syllable structure, lexical prosody, nominal number, locus of marking of nominal possession, and adnominal demonstrative systems. The project uses a fine-grained approach to variable design that assesses the intricacies of each variable, including their behavior under different morphosyntactic and/or semantic conditioning factors (see Bickel 2010 for similar approaches in language typology). Our coding scheme is based on documented cross-linguistic variation in each of these domains (e.g., Maddieson 2013 for syllable structure; Kibort and Corbett 2008 for nominal number; Nichols 1992 for locus of marking; and Maurer 2013 for demonstratives). Additionally, the data collection follows a bottom-up approach that examines which aspects of a given domain of language structure align between languages in contact. Our coding aims to provide answers to the following questions:

(1) *How similar are the Focus and Neighbor Languages with respect to the parameters describing the properties of each linguistic variable?*
(2) *How do similarities between Focus and Neighbor Languages compare to corresponding structural properties of the Benchmark Language within each set?*

Preliminary results obtained using the current sample indicate that our method captures structural similarities between Focus and Neighbor Languages that strongly suggest explanations derived from language contact. That is, we find systematic correspondences between a range of phonological, morphosyntactic, and lexical features of the contact pairs which differ from those attested in the respective Benchmark Languages. Data from six of our sets illustrate those findings in the domain of nominal number. The examples sets were chosen to represent each continental area, that is, Africa, Australia, Eurasia, Papunesia, North America, and South America. The languages in each set, their genealogical affiliations, and a selection of possible contact effects followed by the total number of FL–NL feature matches for each set are given in Table 5. Short descriptions follow.

**Table 5:** Preliminary observations of possible contact effects in selected features of nominal number – number values and their encoding – from six sets (FL: Focus Language; NL: Neighbor Language; BL: Benchmark Language).

SetID	Macro-area	Languages	Number values and their morphosyntactic encoding (selected features)	Total number of FL-NL matches / Total number of potentially relevant features
Set 02	Africa	Bade (bde, Afro-Asiatic; FL) Manga Kanuri (kby, Saharan; NL) Lele (lln, Afro-Asiatic; BL)	Dual number is absent both in the Focus and Neighbor Languages whereas the Benchmark Language has dual number on personal pronouns.	2/8
Set 09	Australia	Mawng (mph, Iwaidjan Proper; FL) Kunbarlang (wlg; Gunwinygyan; NL) Iwaidja (ibd, Iwaidjan Proper; BL)	Both the FL and NL have dual number on personal pronouns only, whereas the dual is fully absent in the BL.	6/6
Set 14	Eurasia	Maltese (mlt, Afro-Asiatic; FL) Sicilian (scn, Indo-European, NL) Gulf Arabic (afb, Afro-Asiatic; BL)	Dual inflections are nearly eroded in the FL and totally lacking in the NL, but they are still productive in the BL.	1/5
Set 15	North America	Yurok (yur, Algic; FL) Karok (kyh, Karok; NL) Naskapi (nsk, Algic; BL)	In the FL and NL, verbs carry special argument indexes when the subject denotes a group (i.e., ‘collective plurals’). The feature is absent in the BL.	3/6
Set 21	Papunesia	Papapana (ppn, Austronesian; FL) Rotokas (roo, North Bougainville; NL) Marshallese (mah, Austronesian; BL)	Dual occurs in the FL, being pervasive in the nominal morphology of the NL, but absent in the BL.	5/21
Set 49	South America	Western Toba (tob, Guaicuruan; FL) Wichí Noctén (mtp, Matacoan; NL) Kadiwéu (kbc, Guaicuruan; BL)	The FL and NL have nominal affixes used to denote groups (aka ‘collective markers’), which are absent in the BL.	5/9

^a^The coding scheme for nominal number consists of 55 distinct variables. In our coding scheme, FL = NL ≠ BL equals ‘1’, while FL = BL ≠ NL equals ‘0’. Thus, for each set, the total number of FL–NL matches is the sum of all instances in which the feature value of the FL and NL are the same while the BL differs. The total number of potentially relevant features is the sum of the ‘1’ and ‘0’ for each set.

One striking pattern emerges from the data presented in Table 5. Focus and Neighbor languages across these six sets are similar to each other by either possessing or lacking one number category (notably the dual or collective plurals), whereas the opposite pattern is found in the Benchmark for each set. While number systems across these sets differ in their morphosyntactic and semantic behaviors, the patterns observed suggest that pairs of Focus and Neighbor languages show similarities by either possessing or lacking typologically less frequent values compared to the respective Benchmarks.

The scenario in which Focus and Neighbor languages share number values missing in the Benchmark is illustrated by Sets 09, 15, 21, and 49. In Set 09, from Northern Australia, both Mawng and Kunbarlang have dual inflections on independent personal pronouns (Kapitonov 2019; Singer 2006), but these are absent in the Benchmark Language Iwaidja. In Set 21, the Focus Language Papanana marks dual number in the collective forms of the articles (Smith-Dennis 2021: 191), similarly to the Neighbor Language Rotokas, in which dual marking is generally pervasive (Robinson 2011), whereas the Benchmark Marshallese lacks dual entirely. Similarly, both Yurok and Karok in Set 15 have verb forms described as “collective plurals” which are used to index that the subject denotes a group of entities (Bright 1957: 88; Garrett 2014: 41–46). These forms are not reported for the Benchmark Naskapi. Finally, in Set 49, both the Focus Language Western Toba and the Neighbor Language Wichí Noctén are reported to have a series of nominal affixes that encode collective and distributive meanings. This type of affixes is not attested in the Benchmark Language Kadiwéu (cf. Carpio 2012: 71–73 for Western Toba; Terraza 2009: 88–93 for Wichí Noctén).

The opposite pattern, where certain number values are attested in the Benchmark, but absent in the Focus and Neighbor languages, is found in Set 02 and Set 14. In Set 02, dual forms are attested in the Benchmark Lele (Frajzyngier 2001), but absent in the Focus Language Bade (Schuh 2005) and in the Neighbor Language Manga Kanuri (Hutchinson 1981). This situation is akin to Set 14, where dual inflections are frequent in the Benchmark Gulf Arabic (Holes 1990) while nearly absent in the Focus Language Maltese (Borg 1994); the Neighbor Language for this set, Sicilian, lacks dual entirely (Bigalke 1997).

Having first described these findings, we now turn to a word of caution. While the fact that a feature is present in two unrelated languages in contact but absent in the Benchmark may be taken as indication of contact-induced change, the absence of a feature in both languages arguably constitutes less clear evidence of contact. In other words, instances when two unrelated languages in contact both lack a given feature which is present in the Benchmark Language (which would correspond to a ‘0–0–1’ in our coding method) would be less indicative of contact-induced change than when both Focus and Neighbor Languages contain a feature absent in the Benchmark (1–1–0 in our coding method). Such a conclusion is warranted especially in cases of typologically frequent properties.11The absence of typologically common features in groups of languages in contact has indeed been used as indication of contact in linguistic areas. For instance, the absence of nasal phonemes in indigenous languages of present-day US/Canada Pacific Northwest (e.g., Thomason 2001: 123), as well as the near total absence of fricatives in most languages of Australia (e.g., Dixon 2001: 67, among others) constitute two of the relevant features in the characterization of contact in those areas of the world. Put simply, one should be cautious to use ‘shared absences’ as the sole indication of contact. Expanding the basis of comparison to more than one Benchmark would seem especially important for better contextualizing shared absence as indication of contact.12We thank one anonymous reviewer and Patience Epps for this important remark. Nevertheless, all things being equal, both ‘shared presence’ and ‘shared absence’ contribute relevant datapoints to the overall assessment of a given contact profile.

It should be noted that the method being proposed here does not preclude more in-depth, comparative analyses of contact effects in individual languages. Rather, our method provides a sample which researchers can use as a starting point for further investigations of contact. It mitigates the issue of similarity due to inheritance by proposing the analysis of maximally unrelated languages. It offers an additional tool for control by proposing the inclusion of a Benchmark Language against which variables should be compared.

The current sampling method thus provides typologists with a testing ground for investigating contact effects on a large scale. Preliminary observations from six sets from across the globe suggest that data collection using this sampling technique allows for the detection of structural similarities between given pairs of Focus and Neighbor Languages. The addition of Benchmark Languages provides a systematic way to further ensure that any similarities uncovered may be due to contact. The next section discusses limitations in the procedure.

### 4.3 Limitations

As hinted above, the current procedure does not preclude detailed areal and historical-comparative analyses of specific cases of contact, all of which remain paramount. Additionally, while successful in generating a sample of languages suitable for the study of contact from a typological perspective, the current method presents limitations, three of which we discuss here. Two limitations concern Benchmark Languages, namely how many Benchmark to consider per set, and the differing degrees of relatedness between Benchmarks and Focus Languages across the sets. The third limitation addressed in this section is the project-specific constraint that requires that we find living experts on the Focus Languages and/or contact scenarios with whom to collaborate.

#### 4.3.1 Number of Benchmark Languages

The current method utilizes a single Benchmark language per set. This choice has the advantage of allowing for the inclusion of languages from small language families in specific sets (e.g., Naduhup). However, it is conceivably less powerful a check than if a greater number of languages were included as Benchmarks for the same contact pair. This choice seemingly introduces some degree of randomness in the selection procedure, which may be difficult to quantify across sets.13One way of lowering the degree of uncertainty associated with the one-Benchmark approach would be to create a dataset for which multiple Benchmarks are only selected for those Focus languages that belong to larger language families were several candidate sister languages exist. While we acknowledge that, in principle, this would decrease the degree of uncertainty associated with some language sets, in this paper, and for the purposes of the larger project, we opted for a conservative approach (i.e., one Benchmark for each sampled contact pair). Needless to say, this solution can be easily implemented in future applications of the sampling method and, in fact, it has already been partially applied in individual case studies within our larger project (see the Alorese-Adang-Lamahalot and Zazaki-Turkish-W. Balochi cases briefly discussed in this section). Despite its limitations, the method proposed here is certainly expandable, and allows individual researchers to select as many Benchmark languages as are available using the same selection criteria outlined in Section 2.3. Nevertheless, we would argue that the procedure as it stands is already sufficient to run a screen-through of potential contact effects within individual contact sets as well as on a global scale.

In addition to the data on number systems presented in Section 4.2, two additional tests suggest that a single Benchmark provides valid control for detecting contact effects. The first test considers the efficacy of having several Benchmarks, whereas the second is an illustration of the method using a well-studied language area, namely the Balkans.

In the context of the project at large, Sinnemäki and Ahola (2022) tested the robustness of having a single Benchmark in two of our sampled sets. The authors used locus of marking of adnominal possession as the linguistic variable in Sets 21 and 26. Set 21 has Alorese (Austronesian) as the FL, Adang (Timor-Alor-Pantar) as the NL and Lamahalot (Austronesian) as the BL, whereas Set 26 has Zazaki (Indo-European) as the FL, Turkish (Turkic) as the NL, and Western Balochi (Indo-European) as the BL. The authors compared adnominal possessive constructions attested in each of the two contact pairs against a larger selection of Benchmarks (i.e., 13 Benchmarks for Set 21, and nine Benchmarks for Set 26). The analyses revealed that using additional Benchmarks that fit the criteria we outline in Section 2 (i.e., neither in contact with the Focus or the Neighbor Language) captures structural convergence between FLs and NLs in very similar ways to the results obtained by using a single Benchmark.

The second illustration focuses on the Balkans, a contact scenario that is not featured in the dataset presented in this paper. Using a set of ‘Balkanisms’ put forward in Joseph (2020), we can evaluate the contact scenario between two core Balkan languages, namely Romanian and Macedonian, with close relatives spoken outside the area. Taking Romanian as a Focus Language, and Macedonian as a Neighbor Language, our procedure would lead us to select Standard Italian as the Benchmark Language. Table 6 showcases how a selection of morphosyntactic features found in both languages compares to analogous constructions in Standard Italian.

**Table 6:** An illustration of the distribution of Balkanisms using Romanian as a Focus Language, Macedonian as a Neighbor Language, and Standard Italian as the sole Benchmark.

Feature	Romanian-FL Source: Cojocaru (2004)	Macedonian-NL Source: Foulon-Hristova (1998)	Italian-BL Source: Maiden and Robustelli (2007)
Future tense based on the verb ‘want’	Yes (p. 141)	Yes (p. 150)	No (pp. 219–221)
Postposed definite article	Yes (p. 42)	Yes (p. 69)	No (ch. 4)
Analytic comparative adjective formations	Yes (pp. 51–52)	Yes (p. 66)	Yes (ch. 16)
Prepositional object marking with pronouns	Yes (p. 55)	Yes (p. 77)	No (p. 96)
Double determination in deixis	Yes (p. 78)	Yes (p. 17)	No (ch. 4, 5)

As the table shows, Romanian and Macedonian pattern similarly while differing from Italian, with the sole exception of ‘Analytic comparative adjective formations’. The key point here is that Standard Italian, the Benchmark in this scenario, differs in almost every aspect from the Focus Language.

Although somewhat simplistic, the comparisons above underscore two important points that relate to the role of the Benchmark in our method. First, since the current sampling technique uses already-established contact scenarios as its starting point (i.e., it is a phenomenon-based sample), this potentially reduces the need for family-wide checks. Secondly, the fact that the Benchmark language tends to be the closest possible relative of the Focus Language increases the likelihood that *systematic differences* between the Focus and Benchmark languages stem from external influence. This is especially clear in cases like the Romanian and Italian examples above, in which the features that set Romanian apart from Italian on the one hand are the same as those that connect Romanian and Macedonian on the other.

While this Balkan scenario would violate the sampling criteria in our method, given that both Romanian and Macedonian are Indo-European, it highlights how effective a systematic comparison with a single Benchmark can be. All things being equal, one would expect that similarities between completely unrelated languages would be even more suggestive of contact, especially when the Focus Language differs systematically from its Benchmark. As mentioned above, the current procedure aims at determining *the extent to which* contact may impact structures of the Focus Language, rather than establishing whether or not contact occurs. We contend that researchers aiming to validate claims of contact-induced change specific to individual linguistic areas and families therein would likely benefit from the systematic analysis of several benchmark languages.

#### 4.3.2 Varying degrees of relatedness between Benchmark and Focus Languages

Because the distribution of languages within families and contact scenarios differs widely across the globe, degrees of relatedness between Focus and Benchmark languages are also uneven across our sampled sets. For instance, a Focus Language in a given set may only have distant relatives (i.e., members of distinct subfamilies), or candidate Benchmark languages may also be in contact with the Neighbor, as discussed for Hup and Yuhup. One way to address these discrepancies across sets is to include a measure of relatedness as an independent variable in statistical analyses. One such measure already used in typology, which could also be applied to this case, is the Diversity Value (Rijkhoff et al. 1993; Rijkhoff and Bakker 1998).

While initially proposed for generating variety samples, using the Diversity Value could serve our purposes here as it would provide a way to quantify the genealogical distance between Focus and Benchmark Languages across sets. The Diversity Value is a numeric measure that considers both the depth and the width of a language family tree. The depth relates to the numbers of levels within a language family, whereas the width refers to the number of nodes across a level. The complexity of the language family tree determines the Diversity Value, which is recursively calculated from the number of levels *under* the top level. Although we propose that the Diversity Values can be relevant for the analyses of typological distributions in the data, they would have no *a priori* role in the procedure of language selection as such, which is why we leave this for future implementation.

#### 4.3.3 Sampling composition and availability of questionnaire respondents

The sampling composition was partly determined by the need for experts who could respond to a sociolinguistic questionnaire we developed for the project at large (Kashima et al. under revision). That is, the sample was often tied to the existence of potential collaborators, mostly authors of reference grammars, or specialists in contact scenarios.14When selecting experts, we strived to include researchers either from the communities under study or at least from universities located in the same country as the contact scenarios. The choice to select local experts stems from our wish to engage with different academic cultures, and with more local perspectives on the language communities under scrutiny. We acknowledge that this choice represents but a tentative step towards building more constructive and equitable relationships with indigenous populations in our field (cf. Charity Hudley et al. 2020; Louie et al. 2017). As a result, it could be argued that the sample presented here perhaps reflects more recent contact dynamics than historical scenarios known to have left an impact on the structures of the languages involved. This is because the questionnaire comprises questions that are better suited to describe the sociolinguistic patterns behind contact between Russian and Udmurt (udm), for instance, than those between Sumerian (sux) and Akkadian (akk), or Classical Nahuatl (nci) and Epigraphic Mayan (emy).

Due to these project-specific goals, consulting with experts also resulted in a few revisions to the composition of the language sample that we present here. For instance, Set 05 initially investigated contact dynamics between the Northern Songhay language Tadaksahak (dsk) and the Afro-Asiatic language Tetserret (tez). After consulting with the expert, we updated the set so that it now investigates contact between speakers of the Northern Songhay language Korandje (kcy) and Algerian Arabic (aao) instead.

Although this project-specific criterion influenced the current sample to a certain degree, the criteria behind the general selection were still the same. Indeed, the fact that replacements prompted by experts were found once again speaks to the efficacy of the method being proposed. This suggests that the procedure is fit to generate samples for typological studies that focus solely on linguistic outcomes of contact as much as it does for investigations of its sociolinguistic aspects.

### 4.4 Current implementation of the procedure

As mentioned above, the sampling technique presented in this paper is part of the research design of a larger project investigating how various sociolinguistic contexts impact around 200 variables in the realms of morphosyntax, phonology, and the lexicon. The dataset presented in Section 3 and Appendix A (in the Supplementary Materials) is a demonstration of the sampling technique as well as the actual source of linguistic data for the project. This section briefly illustrates how the sampling procedure serves the goals of the project at large.

The sampling technique serves three interrelated goals within the larger project. First, the dataset resulting from the sampling procedure enables us to conduct comparisons of contact scenarios from a sociolinguistic perspective. Secondly, the sample serves as the source for data on linguistic variables in the languages of each set (illustrated in Section 4). Thirdly, the sample allows us to study how the sociolinguistic profiles observed in different contact scenarios relate to the patterns uncovered through the comparison of language structures within sets. Achieving these three goals would thus generate a systematic account of linguistic contact, encompassing both its sociolinguistic underpinnings and its potential impact on linguistic structure.

In this design, a sociolinguistic questionnaire probes into contact dynamics between Focus and Neighbor language communities in six different domains: family and kin, ceremonial exchange, daily interactions, labor, knowledge, and trade. Investigating contact in these domains serves as a description of the nature of contact between the Focus and Neighbor communities. The questions in the questionnaire are informed by the literature on contact, psycholinguistics, historical linguistics, second-language acquisition, among others (see Di Garbo et al. 2021). Questions address topics such as the duration and density of contact between Focus and Neighbor languages, social network structures, language attitudes, patterns of language use and transmission, and so forth.

Table 7 provides an overview of the sample composition by six macro-areas as of November 2021, focusing on the match between the contact scenarios identified in the literature (‘Target’) and those for which we were able to identify respondents to our questionnaire (‘Obtained’).

**Table 7:** Questionnaire responses by area (as of November 2021).

Macro-area	Target (*N*)	Obtained (*N*)	Percentage
Africa	08	08	100
Eurasia	13	11	85
Papunesia	09	07	78
Australia	03	02	67
South America	07	04	58
North America	10	05	50
*Total*	50	37	74

As illustrated in Table 7, we attained adequate matches between the number of contact pairs identified in the literature and expert responses for Africa and Eurasia, with a coverage of 100 and 85 percent, respectively. High coverage (between 78 and 58 percent) was also reached for Papunesia, Australia and South America. This result is particularly promising given that these areas tend to be underrepresented in typological research, often due to lack of accessible resources. However, for North America, only 50 percent of the initial candidates for contact pairs yielded questionnaire responses from experts. This observation contrasts with the fact that the area is usually well-covered in typological studies focusing only on linguistic features.

The low targets reached for an area such as North America may reflect the insufficient documentation of sociolinguistic phenomena in Native languages, as discussed in Section 3. The lack of responses to our questionnaire also reveals the degree of language obsolescence in indigenous communities. We note that we are retaining the full-fledged sample for collecting linguistic data regarding our 200 variables (Section 4). However, it follows that the sociolinguistic data come only from those sets for which we established collaborations with experts.

### 4.5 Further applications

The current sampling procedure is applicable to different research goals. While the focus of the current dataset is on contact dynamics between genealogically unrelated languages, the language selection criteria introduced here could also serve investigations of contact between related languages (see Section 4.3 for an illustration using the Balkans). For related languages, generating a sample would only entail reconfiguring the internal composition of the three-language sampling units.

What’s more, the current sample partly depended on the availability of living experts with whom we could collaborate in the sociolinguistic component within the larger project. In Section 4.3 we discuss how this project-specific strategy may have led to an emphasis on more recent contact scenarios. Future studies based solely on reference materials may thus be less constrained in their choice of contact scenarios, possibly permitting the investigation of contact situations for which no sociolinguistic information is available.

Finally, as reiterated throughout this article, this sampling technique is phenomenon-based, stemming from the manual assessment of the contact literature. A possible development of the current method would be to implement an automatized procedure of language selection based on similar design principles. This would enable the researcher to generate possibly larger datasets than the one presented here.

## 5 Concluding remarks

In this paper, we introduced a sampling procedure specifically tailored to studies of language contact from a typological perspective. To our knowledge, this is the first sampling method that draws directly from documented contact scenarios, and one that allows for systematic comparisons of contact effects on a global scale. The technique fulfills key criteria in sampling methods in language typology such as representativeness and independence. It can also be expanded beyond the type of contact scenarios targeted in our project at large (e.g., to address contact between genealogically related languages). Finally, the procedure can be easily re-applied. Indeed, not only did it generate the contact sets that comprise the sample presented in this paper, but also multiple alternatives.

Throughout the article, we also underscored the importance of having a control regarding the issue of genealogy versus inheritance in comparative contact studies. By introducing a systematic way for selecting a benchmark against which to compare changes in a given language, this procedure provides a methodological contribution to the typological study of contact phenomena. While the proposed design cannot provide the ultimate solution to the ‘diffusion versus inheritance’ conundrum, it certainly adds a new set of resources to the typologist’s toolkit for capturing, and thereby assessing, contact signals in the languages of the world. We argue that this newly developed tool may serve as a point of departure for studies of linguistic diversity in general, and of language contact in particular.

**Software and packages:** Figures and maps were generated in R (R Core Team 2019). All maps were made using the ‘lingtypology’ package (Moroz 2017).

## Supplementary Material

Supplementary Material Details

Supplementary Material Details
