# Supplementary material for: A sampling technique for worldwide comparisons of language contact scenarios
Source: Linguist Typol. 2023 Feb 10;27(3):553–89. doi: 10.1515/lingty-2022-0005 (PMC10551743; doi:10.1515/lingty-2022-0005)
Supplement: Supplementary file 2 — Supplementary Material Details [file j_lingty-2022-0005_suppl_002.docx]

**APPENDIX A: The full dataset (language families abbreviations listed in Appendix B)**

| **Language** | **Glottocode** | **ISO** | **Family** | **Type** | **SetID** | **Autotyp Area** | **Sources** |  |
| --- | --- | --- | --- | --- | --- | --- | --- | --- |
| Baïnounk Gubëeher | gube1234 |  | ACG | Focus | 01 | African Savannah |  |  |
| Mandinka | mand1436 | mnk | MDE | Neighbor | 01 | African Savannah | Cobbinah (2010) |  |
| Bijogo | bidy1244 | bjg | ACG | Benchmark | 01 | African Savannah |  |  |
| Bade | bade1248 | bde | AFA | Focus | 02 | African Savannah |  |  |
| Manga Kanuri | mang1399 | kby | SAH | Neighbor | 02 | African Savannah | Ziegelmeyer (2015) |  |
| Lele | lele1276 | lln | AFA | Benchmark | 02 | African Savannah |  |  |
| Mursi | murs1242 | muz | SUR | Focus | 03 | Greater Abyssinia |  |  |
| Hamer-Banna | hame1242 | amf | SMC | Neighbor | 03 | Greater Abyssinia | Güldemann (2018) |  |
| Tennet | tenn1246 | tex | SUR | Benchmark | 03 | Greater Abyssinia |  |  |
| Kambaata | kamb1316 | ktb | AFA | Focus | 04 | Greater Abyssinia |  |  |
| Wolaytta | wola1242 | wal | TNO | Neighbor | 04 | Greater Abyssinia | Treis (2012) |  |
| Xamtanga | xamt1239 | xan | AFA | Benchmark | 04 | Greater Abyssinia |  |  |
| Korandje | kora1291 | kcy | SGY | Focus | 05 | North Africa |  |  |
| Algerian Arabic | alge1239 | aao | AFA | Neighbor | 05 | North Africa | Souag (2010) |  |
| Zarma | zarm1239 | dje | SGY | Benchmark | 05 | North Africa |  |  |
| Nobiin | nobi1240 | fia | NUB | Focus | 06 | North Africa |  |  |
| Beja | beja1238 | bej | AFA | Neighbor | 06 | North Africa | Güldemann (2018); Miller (1996) |  |
| Karko | kark1256 | kko | NUB | Benchmark | 06 | North Africa |  |  |
| Ndebele | nort2795 | nde | ACG | Focus | 07 | Southern Africa |  |  |
| Tjwao (Northern Tshwa) | tsoa1238 | hio | KKW | Neighbor | 07 | Southern Africa | Güldemann & Fehn (2017) |  |
| Gyele | gyel1242 | gyi | ACG | Benchmark | 07 | Southern Africa |  |  |
| Langi/Rangi | lang1320 | lag | ACG | Focus | 08 | Southern Africa |  |  |
| Alagwa | alag1248 | wbj | AFA | Neighbor | 08 | Southern Africa | Gibson & Marten (2019) |  |
| Zulu | zulu1248 | zul | ACG | Benchmark | 08 | Southern Africa |  |  |
| Mawng | maun1240 | mph | IWA | Focus | 09 | North Australia |  |  |
| Kunbarlang | kunb1251 | wlg | GUN | Neighbor | 09 | North Australia | Singer (2018), Singer & Harris (2016) |  |
| Iwaidja | iwai1244 | ibd | IWA | Benchmark | 09 | North Australia |  |  |
| Burarra | bura1267 | bvr | MNG | Focus | 10 | North Australia |  |  |
| Djinang | djin1253 | dji | PMN | Neighbor | 10 | North Australia | Vaughan (2018) |  |
| Djeebbana | djee1236 | djj | MNG | Benchmark | 10 | North Australia |  |  |
| Yawuru | yawu1244 | ywr | NYY | Focus | 11 | South Australia |  |  |
| Karadjeri | kara1476 | gbd | PMN | Neighbor | 11 | South Australia | Evans (2003) |  |
| Bardi | bard1255 | bcj | NYY | Benchmark | 11 | South Australia |  |  |
| Gascon | occi1239 | oci | IEU | Focus | 13 | Europe |  |  |
| Basque | basq1248 | eus | BAS | Neighbor | 13 | Europe | Haase (1992) |  |
| Ligurian | ligu1248 | lij | IEU | Benchmark | 13 | Europe |  |  |
| Maltese | malt1254 | mlt | AFA | Focus | 14 | Europe |  |  |
| Sicilian | sici1248 | scn | IEU | Neighbor | 14 | Europe | Sansò (2011) |  |
| Gulf Arabic | gulf1241 | afb | AFA | Benchmark | 14 | Europe |  |  |
| Yurok | yuro1248 | yur | ALG | Focus | 15 | California |  |  |
| Karok | karo1304 | kyh | KYH | Neighbor | 15 | California | Conathan (2004) |  |
| Naskapi | nask1242 | nsk | ALG | Benchmark | 15 | California |  |  |
| Cupeno | cupe1243 | cup | UAZ | Focus | 16 | California |  |  |
| Tipai/Jamul Tiipay | kumi1248 | dih | CCY | Neighbor | 16 | California | Hinton (1991) |  |
| Huarijio | huar1255 | var | UAZ | Benchmark | 16 | California |  |  |
| Eastern Canadian Inuktitut | east2534 | ike | ESK | Focus | 17 | Eastern North America |  |  |
| Northern East Cree | nort1552 | crl | ALG | Neighbor | 17 | Eastern North America | Barger (1979) |  |
| Tunumiisiut | tunu1234 |  | ESK | Benchmark | 17 | Eastern North America |  |  |
| Choctaw | choc1276 | cho | MUS | Focus | 18 | Eastern North America |  |  |
| Biloxi | bilo1248 | bll | SUX | Neighbor | 18 | Eastern North America | Kaufman (2014, 2019, 2020) |  |
| Creek | cree1270 | mus | MUS | Benchmark | 18 | Eastern North America |  |  |
| Garifuna | gari1256 | cab | AWK | Focus | 19 | Mesoamerica |  |  |
| French | stan1290 | fra | IEU | Neighbor | 19 | Mesoamerica | Michelle Ocasio (pers. comm.) |  |
| Ashéninka Perené | ashe1272 | prq | AWK | Benchmark | 19 | Mesoamerica |  |  |
| Pipil | pipi1250 | ppl | UAZ | Focus | 20 | Mesoamerica |  |  |
| Kaqchikel | kaqc1270 | cak | MYN | Neighbor | 20 | Mesoamerica | Campbell (1985) |  |
| Yaqui | yaqu1251 | yaq | UAZ | Benchmark | 20 | Mesoamerica |  |  |
| Papapana | papa1265 | ppn | AUN | Focus | 21 | Oceania |  |  |
| Rotokas | roto1249 | roo | NOL | Neighbor | 21 | Oceania | Smith-Dennis (2016, 2021) |  |
| Marshallese | mars1254 | mah | AUN | Benchmark | 21 | Oceania |  |  |
| Alorese | alor1247 | aol | AUN | Focus | 22 | Oceania |  |  |
| Adang | adan1251 | adn | TLP | Neighbor | 22 | Oceania | Klamer (2012); Moro (2018, 2019) |  |
| Lewoingu Lamaholot | lama1277 | slp | AUN | Benchmark | 22 | Oceania |  |  |
| (North) Tehuelche | tehu1242 | teh | CHN | Focus | 23 | Andean |  |  |
| Mapuche | mapu1245 | arn | ARC | Neighbor | 23 | Andean | Virkel (2005) |  |
| Selk'nam | onaa1245 | ona | CHN | Benchmark | 23 | Andean |  |  |
| Cusco Quechua | cusc1236 | qvc | QUE | Focus | 24 | Andean |  |  |
| Matsigenka | mach1267 | mcb | AWK | Neighbor | 24 | Andean | Emlen (2020) |  |
| South Bolivian Quechua | sout2991 | quh | QUE | Benchmark | 24 | Andean |  |  |
| Gardabani Neo-Aramaic | boht1238 | bhn | AFA | Focus | 25 | Greater Mesopotamia |  |  |
| Northern/Kurmanji Kurdish | nort2641 | kmr | IEU | Neighbor | 25 | Greater Mesopotamia | Khan (2018) |  |
| Modern Hebrew | hebr1245 | heb | AFA | Benchmark | 25 | Greater Mesopotamia |  |  |
| Zazaki/Zaza | lazz1240 | zza | IEU | Focus | 26 | Greater Mesopotamia |  |  |
| Turkish | nucl1301 | tur | TUK | Neighbor | 26 | Greater Mesopotamia | Haig (2017); Lacroix (2009) |  |
| Western Balochi | west2368 | bgn | IEU | Benchmark | 26 | Greater Mesopotamia |  |  |
| Santali | sant1410 | sat | ATC | Focus | 27 | Indic |  |  |
| Bengali | beng1280 | ben | IEU | Neighbor | 27 | Indic | Peterson (2010) |  |
| Gata' | gata1239 | gaq | ATC | Benchmark | 27 | Indic |  |  |
| Kupwar Marathi | mara1378 | mar | IEU | Focus | 28 | Indic |  |  |
| Kupwar Kannada | nucl1305 | kan | DRA | Neighbor | 28 | Indic | Kulkarni-Joshi (2016) |  |
| Chakma | chak1266 | ccp | IEU | Benchmark | 28 | Indic |  |  |
| Wutunhua/Wutun | wutu1241 | wuh | STB | Focus | 29 | Inner Asia |  |  |
| Bonan | bona1250 | peh | MLK | Neighbor | 29 | Inner Asia | Janhunen (2007) |  |
| Cantonese | cant1236 |  | STB | Benchmark | 29 | Inner Asia |  |  |
| Xibe | xibe1242 | sjo | TSI | Focus | 30 | Inner Asia |  |  |
| Uighur | uigh1240 | uig | TUK | Neighbor | 30 | Inner Asia | Pakendorf (2010) |  |
| Even | even1260 | eve | TSI | Benchmark | 30 | Inner Asia |  |  |
| Central Siberian Yupik | nauk1242 | ess | ESK | Focus | 31 | North Coast Asia |  |  |
| Chukchi | chuk1273 | ckt | CHU | Neighbor | 31 | North Coast Asia | de Reuse (1994) |  |
| Central Alaskan Yupik | cent2127 | esu | ESK | Benchmark | 31 | North Coast Asia |  |  |
| Northern/Tundra Yukaghir | nort2745 | ykg | YKG | Focus | 32 | North Coast Asia |  |  |
| Sakha/Yakut | yaku1245 | sah | TUK | Neighbor | 32 | North Coast Asia | Pupynina and Aralova (2021) |  |
| Southern/Kolyma Yukaghir | sout2750 | yux | YKG | Benchmark | 32 | North Coast Asia |  |  |
| Muak Sa-Aak | tail1246 | tlq | ATC | Focus | 33 | Southeast Asia |  |  |
| Lü/Tai Lue | luuu1242 | khb | TAI | Neighbor | 33 | Southeast Asia | Hall (2010, 2014); Jenny (2015) |  |
| Pnar | pnar1238 | pbv | ATC | Benchmark | 33 | Southeast Asia |  |  |
| Burmese | nucl1310 | mya | STB | Focus | 34 | Southeast Asia |  |  |
| Mon | monn1252 | mnw | ATC | Neighbor | 34 | Southeast Asia | Hall (2010, 2014), Jenny (2015) |  |
| Kurtokha/Kurtöp | kurt1248 | xkz | STB | Benchmark | 34 | Southeast Asia |  |  |
| Aleut | aleu1260 | ale | ESK | Focus | 35 | Alaska-Oregon |  |  |
| Eyak | eyak1241 | eya | AET | Neighbor | 35 | Alaska-Oregon | Leer (1991), Enrico (2004) |  |
| Central Alaskan Yupik | cent2127 | esu | ESK | Benchmark | 35 | Alaska-Oregon |  |  |
| Nuxalk/Bella Coola | bell1243 | blc | SLS | Focus | 36 | Alaska-Oregon |  |  |
| Kwak'wala | kwak1269 | kwk | WAK | Neighbor | 36 | Alaska-Oregon | Beck (2000) |  |
| Okanagan | okan1243 | oka | SLS | Benchmark | 36 | Alaska-Oregon |  |  |
| Towa | jeme1245 | tow | KWT | Focus | 37 | Basin and Plains |  |  |
| Eastern Keres | east1472 | kee | KES | Neighbor | 37 | Basin and Plains | Shaul & Ortman  (2014) |  |
| Kiowa | kiow1266 | kio | KWT | Benchmark | 37 | Basin and Plains |  |  |
| Hopi | hopi1249 | hop | UAZ | Focus | 38 | Basin and Plains |  |  |
| Zuni | zuni1245 | zun | ZUN | Neighbor | 38 | Basin and Plains | Shaul & Ortman (2014) |  |
| Ute-Southern Pauite | utes1238 | ute | UAZ | Benchmark | 38 | Basin and Plains |  |  |
| Huli | huli1244 | hui | NTN | Focus | 39 | Interior New Guinea |  |  |
| Duna | duna1248 | duc | DUN | Neighbor | 39 | Interior New Guinea | San Roque & Loughnane (2012) |  |
| South Awyu | sout2941 | aws | NTN | Benchmark | 39 | Interior New Guinea |  |  |
| Ipili | ipil1240 | ipi | NTN | Focus | 40 | Interior New Guinea |  |  |
| Hewa | hewa1241 | ham | SEP | Neighbor | 40 | Interior New Guinea | Fedden (2011) |  |
| Mauwake | mauw1238 | mhl | NTN | Benchmark | 40 | Interior New Guinea |  |  |
| Kwoma | kwom1262 | kmo | SEP | Focus | 41 | North Coast New Guinea |  |  |
| Manambu | mana1298 | mle | NDU | Neighbor | 41 | North Coast New Guinea | Ross (1996) |  |
| Awtuw | awtu1239 | amp | SEP | Benchmark | 41 | North Coast New Guinea |  |  |
| Paluai | balu1257 | blq | AUN | Focus | 42 | North Coast New Guinea |  |  |
| Tok Pisin | tokp1240 | tpi | IEU | Neighbor | 42 | North Coast New Guinea | Reesink & Dunn (2017) |  |
| Mono-Alu | mono1273 | mte | AUN | Benchmark | 42 | North Coast New Guinea |  |  |
| Coastal Marind | nucl1622 | mrz | ANM | Focus | 43 | Southern New Guinea |  |  |
| Marori | moro1289 | mok | MRR | Neighbor | 43 | Southern New Guinea | Evans (2012); Olsson (2017) |  |
| Jaqay/Yakhai | yaqa1246 | jaq | ANM | Benchmark | 43 | Southern New Guinea |  |  |
| Kala Lagaw Ya | kala1377 | mwp | PMN | Focus | 44 | Southern New Guinea |  |  |
| Meryam Mir/Meri | meri1244 | ulk | ETF | Neighbor | 44 | Southern New Guinea | Evans (2012); Evans et al. (2018) |  |
| Umpila | umpi1239 | ump | PMN | Benchmark | 44 | Southern New Guinea |  |  |
| Nen | nenn1238 | nqn | YAM | Focus | 45 | Southern New Guinea |  |  |
| Idi-Taeme | idii1243 | idi | PHR | Neighbor | 45 | Southern New Guinea | Evans (2012); Evans et al. (2018) |  |
| (Ngkontar) Ngkolmpu | ngka1235 | kcd | YAM | Benchmark | 45 | Southern New Guinea |  |  |
| Kuikuro-Kalapalo | kuik1246 | kui | CRB | Focus | 46 | Northeast South America |  |  |
| Trumai | trum1247 | tpy | TRM | Neighbor | 46 | Northeast South America | Franchetto (2011) |  |
| Pará Arára | para1310 | aap | CRB | Benchmark | 46 | Northeast South America |  |  |
| Mekens | saki1248 | skf | TPN | Focus | 47 | Northeast South America |  |  |
| Aikanã | aika1237 | tba | AIK | Neighbor | 47 | Northeast South America | Crevels & van der Voort (2008) |  |
| Akuntsu | akun1241 | aqz | TPN | Benchmark | 47 | Northeast South America |  |  |
| Yuhup | yuhu1238 | yab | NHP | Focus | 48 | Northeast South America |  |  |
| Macuna | macu1260 | myy | TCA | Neighbor | 48 | Northeast South America | Aikhenvald (2011) |  |
| Nadëb | nade1244 | mbj | NHP | Benchmark | 48 | Northeast South America |  |  |
| Western Toba | toba1269 | tob | GUA | Focus | 49 | Southest South America |  |  |
| Wichí Noctén | wich1262 | mtp | MTC | Neighbor | 49 | Southest South America | Messineo (2011) Messineo & Tacconi (2017), Vidal & Braunstein (2020) |  |
| Kadiwéu | kadi1248 | kbc | GUA | Benchmark | 49 | Southest South America |  |  |
| Nivaclé | niva1238 | cag | MTC | Focus | 50 | Southest South America |  |  |
| Enxet Sur | sout2989 | enx | LMC | Neighbor | 50 | Southest South America | Campbell (2013) |  |
| Chorote | iyoj1235 | crt | MTC | Benchmark | 50 | Southest South America |  |  |

# **Appendix B: Language families represented in the sample in alphabetical order by the abbreviation**

| ACG | Atlantic-Congo |
| --- | --- |
| AET | Athabaskan-Eyak-Tlingit |
| AFA | Afro-Asiatic |
| AIK | Aikanã |
| ALG | Algic |
| ANM | Anim |
| ARU | Araucanian |
| ATC | Austroasiatic |
| AUN | Austronesian |
| AWK | Arawak |
| BAS | Basque |
| CCY | Cochimi-Yuman |
| CNN | Chonan |
| CHU | Chukotko-Kamchatkan |
| CRB | Cariban |
| DRA | Dravidian |
| DUN | Duna |
| ESK | Eskimo-Aleut |
| ETF | Eastern Trans-Fly |
| GUA | Guaicuruan |
| GGN | Gunwinyguan |
| IEU | Indo-European |
| IWA | Iwaidjan |
| KES | Keresan |
| KKW | Khoe-Kwadi |
| KTN | Kiowa-Tanoan |
| KYH | Karok |
| LOY | Lengua-Mascoy |
| MDE | Mande |
| MLK | Mongolic-Khitan |
| MNG | Maningridan |
| MRR | Marori |
| MTC | Matacoan |
| MUS | Muskogean |
| MYN | Mayan |
| NDU | Ndu |
| NHP | Naduhup |
| NOL | North Bougainville |
| NTN | Nuclear-Trans-New-Guinea |
| NUB | Nubian |
| NYY | Nyulnyulan |
| PHR | Pahoturi |
| PMN | Pama-Nyungan |
| QUE | Quechuan |
| SAH | Saharan |
| SEP | Sepik |
| SGY | Songhay |
| SLS | Salishan |
| SMC | South Omotic |
| STB | Sino-Tibetan |
| SUR | Surmic |
| SUX | Siouan |
| TAI | Tai-Kadai |
| TCA | Tucanoan |
| TLP | Timor-Alor-Pantar |
| TNO | Ta-Ne-Omotic |
| TPN | Tupian |
| TRM | Trumai |
| TSI | Tungusic |
| TUK | Turkic |
| UAZ | Uto-Aztecan |
| WAK | Wakashan |
| YAM | Yam |
| YKG | Yukaghir |
| ZUN | Zuni |

**References**

Aikhenvald, Alexandra Y. 2011. Areal features and linguistic areas: Contact-induced change and geographical typology. In Osamu Hieda and Christa König & Hirosi Nakagawa (eds.), *Geographical typology and linguistic areas: With special reference to Africa*, 13–39. Amsterdam/Philadelphia: John Benjamins.

Beck, David. 2000. Grammatical convergence and the genesis of diversity in the Northwest Coast Sprachbund. *Anthropological Linguistics* 42(2). 1 – 67.

Campbell, Lyle. 1985. *The Pipili language of El Salvador*. Berlin: Mouton de Gruyter.

Campbell, Lyle. 2013. Language contact and linguistic change in the Chaco. In Ana Suelly Arruda Câmara Cabral & Jorge Domingues Lopes (eds.) *Encontro internacional arqueologia e linguística histórica das línguas indígenas sulamericanas*. Special issue of *Revista Brasileira de Linguística Antropológica* 5(2): 259–291.

Cobbinah, Alexander. 2010. The Casamance as an area of intense language contact: The case of Baïnounk Gubaher*. Journal of Language Contact* 3. 175–202.

Conathan, Lisa. 2004. *Linguistic ecology of Northwestern California: Contact, functional convergence and dialectology*. PhD dissertation, University of California, Berkeley.

de Reuse, Willem Joseph. 1994. *Siberian Yupik Eskimo: The language and its contacts with Chukchi*. (Studies in Indigenous Languages of the Americas.) Salt Lake City: University of Utah Press.

Emlen, Nicholas Q. 2020. *Language, coffee and migration on an Andean-Amazonian frontier*. Tuscon: The University of Arizona Press.

Evans, Nicholas. 2012. Even more diverse than we had thought: The multiplicity of Trans-Fly languages. In Nicholas Evans & Marian Klamer (eds.), *Melanesian languages on the edge of Asia: Challenges for the 21st Century*, 109–149. Honolulu: University of Hawaii Press.

Evans, Nicholas (ed.). 2003. *The non-Pama-Nyungan languages of northern Australia: Comparative studies of the continent's most linguistically complex region.* Canberra: Pacific Linguistics.

Evans, Nicholas, Wayan Arka, Matthew Carroll, Yun Jung Choi, Christian Döhler, Volker Gast, Eri Kashima, Emil Mittag, Bruno Olsson, Kyla Quinn, Dineke Schokkin, Philip Tama, Charlotte van Tongeren & Jeff Siegel. 2018. The languages of Southern New Guinea. In Bill Palmer (ed.), *Papuan languages and linguistics*, 641–894. Berlin: Mouton.

Fedden, Sebastian O. 2011. *A grammar of Mian*. Berlin: Mouton de Gruyter.

Franchetto, Bruna. 2011. Evidências linguísticas para o entendimento de uma sociedade multilíngue: O Alto Xingu [Linguistic evidence for our understanding of multilingual societies: The Alto Xingu]. In Bruna Franchetto (ed.), *Alto Xingu: Uma sociedade multilíngue*, 3-38. Rio de Janeiro: Museu do Indio- FUNAI.

Gibson, Hannah & Lutz Marten. 2019. Probing the interaction of language contact and internal innovation: four case studies of morphosyntactic change in Rangi. *Studies In African Linguistics*, 48 (2019). 63–92.

Güldemann, Tom. 2018. Areal linguistics beyond contact, and linguistic areas of Arabia. In Tom Güldemann (ed.), *The languages and linguistics of Africa*, 448–545. Berlin: Mouton de Gruyter.

Güldemann, Tom & Anne-Maria Fehn. 2017. The Kalahari Basin area as a “Sprachbund” before the Bantu expansion. In Raymond Hickey (ed.), *The Cambridge handbook of areal linguistics*, 500–526. Cambridge: Cambridge University Press.

Haase, Martin. 1992. *Sprachkontakt und Sprachwandel im Baskenland: Die Einflüsse des Gaskognischen und Französischen auf das Baskische*. Hamburg: Buske.

Haig, Geoffrey. 2017. Western Asia: East Anatolia as a Transition Zone. In Hickey, Raymond (ed.), *The Cambridge handbook of areal linguistics*, 396–423. Cambridge: Cambridge University Press.

Hall, Elizabeth. 2014. An analysis of Muak Sa-Aak tone. *Journal of the Southeast Asian Linguistics Society*. 7. 1–10.

Hall, Elizabeth. 2010. *A Phonology of Muak Sa-aak*. MA thesis, Chiang Mai: Payap University.

Janhunen, Juha. 2007. The changing faces of Amdo Qinghai. *Studia Orientalia* 101. 501-510.

Jenny, Mathias. 2015. Modern Mon. In Paul Sidwell & Mathias Jenny (eds.), *The handbook of Austroasiatic Languages*, 553–600. Leiden: Brill.

Kaufman, David. 2020. The Lower Mississippi Valley as a linguistic area. In Grant, Anthony P. (ed.), *The Oxford handbook of language contact*. DOI:10.1093/oxfordhb/9780199945092.013.39.

Kaufman, David. 2019. *Clues to Lower Mississippi Valley histories: Language, archaeology, ethnography.* Lincoln: University of Nebraska Press.

Kaufman, David. 2014. *The Lower Mississippi Valley as a language area*. PhD dissertation, University of Kansas.

Khan, Geoffrey. 2018. The Neo-Aramaic dialects of western Iran. In Geoffrey Haig & Geoffrey Khan (eds.) *The languages and linguistics of Western Asia: An areal perspective,* 481–532. Berlin, Boston: De Gruyter Mouton. https://doi.org/10.1515/9783110421682-015

Klamer, Marian. 2012. Papuan-Austronesian language contact: Alorese from an areal perspective. In Marian Klamer & Nicholas Evans (eds.), *Melanesian languages on the Edge of Asia: Challenges for the 21th Century*, 72–108. Honolulu, HI: University of Hawaiʻi Press.

Kulkarni-Joshi, Sonal. 2016. Forty years of language contact and change in Kupwar: A critical assessment of the intertranslatability model. *Journal of South Asian Languages and Linguistics*, 3(2), 147–174. <https://doi.org/10.1515/jsall-2016-0008>.

Lacroix, René. 2009. Laz relative clauses in a typological and areal perspective, In Peter K. Austin, Oliver Bond, Monik Charette, David Nathan & Peter Sells (eds.), *Language Documentation and Linguistic Theory 2*, London: SOAS. 10

Leer, Jeff. 1991. Evidence for a Northern Northwest Coast language area: Promiscuous number marking and periphrastic possessive constructions in Haida, Eyak, and Aleut. *International Journal of American Linguistics* 57. 158–193.

Messineo, Cristina. 2011. Aproximación tipológica a las lenguas indígenas del Gran Chaco. Rasgos compartidos entre toba (familia guaycurú) y maká (familia mataco-mataguayo). *Indiana* 28. 183–225.

Messineo, Cristina & Temis Lucía Tacconi. 2017. Problemas y desafios de la traducción de las Lenguas Indígenas: Los casos Toba y Maká de la región del Gran Chaco (Argentina y Paraguay). *Cadernos de Tradução* 37. 92–116.

Miller, Chatherine. 1996. Nubien, berbère et beja : notes sur trois langues vernaculaires non arabes de l’Égypte contemporaine. *Égypte/Monde arabe [en ligne]*. 411–431. DOI: 10.4000/ema.1960.

Moro, Francesca. 2018. The plural word hire in Alorese: Contact-induced change from neighboring Alor-Pantar Languages. *Oceanic Linguistics* 57(1). 178–198.

Moro, Francesca. 2019. Loss of morphology in Alorese (Austronesian): Simplification in adult language contact*. Journal of Language Contact* 12(2). 378–403

Olsson, Bruno. 2017. *The Coastal Marind language*. PhD dissertation, Nanyang Technological University.

Pakendorf, Brigitte. 2010. Contact in Siberian Languages. In Hickey, Raymond (ed.) *The Handbook of Language Contact*, 714–737. Oxford: Blackwell Publishing.

Peterson, John. 2010. Language contact in Jharkhand: Linguistic convergence between Munda and Indo-Aryan in eastern-central India. *Himalayan Linguistics* 9(2). 56–86.

Pupynina, Maria & Natalia Aralova. 2021. Lower Kolyma multilingualism: Historical setting and sociolinguistic trend. *International Journal of Bilingualism*. 25(4) 1081–1101

Reesink, Ger & Michael Dunn. 2017. Contact phenomena in Austronesian and Papuan languages. In Bill Palmer (ed.) *The languages and linguistics of the New Guinea Area: A comprehensive guide*. 939–985. Berlin, Boston: De Gruyter Mouton. <https://doi.org/10.1515/9783110295252-009>.

Ross, Malcolm D. 1996. Contact-Induced Change and the Comparative Method: Cases from Papua New Guinea. In Mark Durie and Malcolm Ross (eds.), *The comparative method reviewed: regularity and irregularity in language change*. 180-217. New York/Oxford: Oxford: Oxford University Press.

San Roque, Lila & Robyn Loughnane. 2012. The New Guinea Highlands evidentiality area. *Linguistic Typology* 16. 10.1515/lity-2012-0003.

Sansò, Andrea. 2011. Mediterranean languages. In Kortmann, Bernd & Johan van der Auwera (eds.) *The languages and linguistics of Europe: A comprehensive Guide*. 341–356. Berlin: Mouton de Gruyter.

Shaul, David L. & Scott G. Ortman. 2014. *A prehistory of Western North America: The Impact of Uto-Aztecan languages*. Albuquerque: UNM Press.

Singer, Ruth. 2018. A small speech community with many small languages: The role of receptive multilingualism in supporting linguistic diversity at Warruwi Community (Australia). *Language & Communication* 62. 102–118.

Singer, Ruth & Salome Harris. 2016. What practices and ideologies support small-scale multilingualism? A case study of unexpected language survival in an Australian Indigenous community. *International Journal of the Sociology of Language* 241, 163–208.

Smith-Dennis, Ellen. 2016. Contact-induced change in a highly endangered language of Northern Bougainville. *Australian Journal of Linguistics* 36:3. 369–405, DOI: 10.1080/07268602.2015.1134300

Smith-Dennis, Ellen. 2021. *A grammar of Papapana*. Berlin, Boston: De Gruyter Mouton.

Souag, M. Lameen. 2010. *Grammatical contact in the Sahara: Arabic, Berber, and Songhay in Tabelbala and Siwa*. PhD dissertation: University of London.

Treis, Yvonne. 2012. Switch-reference and Omotic-Cushitic language contact in southwest Ethiopia. *Journal of Language Contact* 5(1) 80–116.

Vaughan, Jill. 2018. “We talk in saltwater words”: Dimensionalisation of dialectal variation in multilingual Arnhem Land. *Language & Communication* 62. 119–132.

Vidal, Alejandra & José Braunstein. 2020. The southern plains and the continental tip. In Tom Güldemann, Patrick McConvell & Richard A. Rhodes (eds.) *The Language of Hunter-Gatherers.* 641–669. Cambridge: Cambridge University Press.

Virkel, Ana 2005. La conversación en comunidades mapuche-tehuelches. Contacto dialectal e interculturalidad/Conversation in Mapuche-Tehuelche Communities. Dialectical Contact and Interculturality. *Anclajes*, 9(9). 263–280.

Ziegelmeyer, Georg. 2015. Areal diffusion in the Chadic-Kanuri contact zone. Paper presented at the Workshop “Areal phenomena in northern sub-Sahara Africa” at the 8th World Congress of African Linguistics, Kyoto University, 20–24 August.
